# Unravelling the Intrinsic Functional Organization of the Human Striatum: A Parcellation and Connectivity Study Based on Resting-State fMRI

**DOI:** 10.1371/journal.pone.0106768

**Published:** 2014-09-09

**Authors:** Wi Hoon Jung, Joon Hwan Jang, Jin Woo Park, Euitae Kim, Eun-Hoe Goo, Oh-Soo Im, Jun Soo Kwon

**Affiliations:** 1 Institute of Human Behavioral Medicine, Seoul National University-Medical Research Center, Seoul, South Korea; 2 Department of Psychiatry, Seoul National University College of Medicine, Seoul, South Korea; 3 Department of Neuropsychiatry, Seoul National University Bundang Hospital, Seongnam, South Korea; 4 Department of Radiological Science, Cheong-ju University, Cheongju, South Korea; 5 Department of Diagnostic Radiology, Seoul National University Hospital, Seoul, South Korea; 6 Deparment of Brain & Cognitive Sciences, College of Natural Sciences, Seoul National University, Seoul, South Korea; Max Planck Institute for Human Cognitive and Brain Sciences, Germany

## Abstract

As the main input hub of the basal ganglia, the striatum receives projections from the cerebral cortex. Many studies have provided evidence for multiple parallel corticostriatal loops based on the structural and functional connectivity profiles of the human striatum. A recent resting-state fMRI study revealed the topography of striatum by assigning each voxel in the striatum to its most strongly correlated cortical network among the cognitive, affective, and motor networks. However, it remains unclear what patterns of striatal parcellation would result from performing the clustering without subsequent assignment to cortical networks. Thus, we applied unsupervised clustering algorithms to parcellate the human striatum based on its functional connectivity patterns to other brain regions without any anatomically or functionally defined cortical targets. Functional connectivity maps of striatal subdivisions, identified through clustering analyses, were also computed. Our findings were consistent with recent accounts of the functional distinctions of the striatum as well as with recent studies about its functional and anatomical connectivity. For example, we found functional connections between dorsal and ventral striatal clusters and the areas involved in cognitive and affective processes, respectively, and between rostral and caudal putamen clusters and the areas involved in cognitive and motor processes, respectively. This study confirms prior findings, showing similar striatal parcellation patterns between the present and prior studies. Given such striking similarity, it is suggested that striatal subregions are functionally linked to cortical networks involving specific functions rather than discrete portions of cortical regions. Our findings also demonstrate that the clustering of functional connectivity patterns is a reliable feature in parcellating the striatum into anatomically and functionally meaningful subdivisions. The striatal subdivisions identified here may have important implications for understanding the relationship between corticostriatal dysfunction and various neurodegenerative and psychiatric disorders.

## Introduction

The basal ganglia comprise the striatum, pallidum, substantia nigra, and subthalamic nucleus. Tract-tracing studies suggest that the basal ganglia are connected to different cortical and subcortical regions, forming multiple parallel cortico–striato–thalamo–cortical (CSTC) loops that can be functionally distinguished (cognitive, affective, and motor processing) by the specific cortical, striatal, and thalamic areas that belong to them [1]. CSTC dysfunction has been implicated in various neurological and neuropsychiatric disorders associated with specific patterns of cognitive, affective, and motor impairments. Thus, exploring the functional topography of CSTC projections is important for understanding the relationship between CSTC dysfunction and various clinical disorders and for developing appropriate treatment.

The striatum is the main input structure of the basal ganglia. In primates, it is anatomically divided by the internal capsule into the caudate and putamen, and the caudate can further be subdivided into the head, body, and tail. The functional distinctions in these striatal subdivisions have been the focus of current research [2]. Although there is no clear anatomical division between the caudate and putamen in rodents, the striatum is functionally and anatomically divided into the dorsal striatum (caudate and putamen) and ventral striatum (nucleus accumbens) in both rodents and primates. For example, whereas the dorsal striatum is closely connected to the motor cortex and the dorsolateral frontal cortex (DLPFC), the key regions for motor control and cognitive function, respectively, the ventral striatum is closely connected to the medial and orbital frontal cortex (OFC), the key regions for reward processing and decision-making, respectively [3].

Researchers have recently attempted to characterize striatal subdivisions and circuitry in humans based on the pattern of anatomical and functional connectivity (FC) revealed by non-invasive brain imaging techniques. For example, many diffusion tensor imaging (DTI) [4]–[9] and resting-state fMRI (rs-fMRI) studies [10]–[12] confirmed the segregation of CSTC connections, showing links from specific striatal areas to different cortical areas.

Recently, the FC approach using rs-fMRI data has been primarily used to investigate distinct functional communities in the brain, called resting-state networks, given that functionally connected areas have related spontaneous fluctuations in the blood oxygen level-dependent (BOLD) signal intensity [13]–[15]. Indeed, this approach can be used as a complementary tool to investigate organizational patterns not captured by other modalities. For example, Di Martino et al. [10] performed seed-to-voxel FC analyses using striatal seeds based on results from a meta-analysis of task-based functional studies [16]. They revealed distinct functional connections (cognitive, affective, and motor systems) to striatal seed regions. However, this study does not provide the answer to a major question regarding the organization of intrinsic striato-cortical networks (i.e., the functional subdivision pattern of the striatum at rest). To address this issue, Barnes et al. [11] and Kim et al. [17] recently employed modularity optimization methods in graph theory terms and multilevel spatial independent component analysis, suggesting at least three modules in the striatum and 31 independent components for the basal ganglia and thalamus. Other studies have used clustering analysis, an increasingly popular and validated method, to parcellate the supplementary motor area (SMA) [18], Broca's area [19], insula [20], OFC [21], cerebellum [22], and cerebral cortex [23]. A recent study used a clustering-based approach to identify the intrinsic organization of the striatum by assigning each voxel in the striatum to its most correlated cerebral network (using 7- and 17-network parcellations identified by Yeo et al. [23]). However, this raises a further question; what would the striatal parcellation look like if you simply performed the unsupervised clustering without subsequent assignment to cortical networks? To address this issue, in this study we employed the *K*-means clustering algorithm to parcellate the striatal regions (i.e., caudate and putamen) according to similarity of FC in different cluster solutions (*K* = 2–10) without any anatomically or functionally defined cortical targets. This approach may help identify natural subsets of intrinsic functional networks in the striatum.

The aim of this study was to confirm previous results regarding functional subdivisions in the human striatum by using a clustering algorithm without any predefined cerebral network targets. First, we subdivided the striatum based on its FC with other whole-brain regions during the resting-state using unsupervised clustering techniques. Second, we explored FC maps between each resulting subregion and other brain regions using both the seed-to-voxel and region-of-interest (ROI)-to-ROI methods. Finally, we subdivided the striatum based on its FC with other brain regions belonging to CSTC loops and then calculated FC maps using an ROI-to-ROI approach.

## Materials and Methods

### Ethics statements

All participants (all right-handed healthy adults, age 19–46 years) gave written informed consent prior to participation. The study was approved by the Institutional Review Boards of the Seoul National University Hospital.

### Participants

Fifty-nine right-handed subjects [33 males, mean age (*SD*), 25.81 (6.45) years] were included in this study. All data in the present analysis were collected in imaging studies in large cohorts of individuals with psychiatric disorders, including obsessive-compulsive disorder and schizophrenia, and controls. All subjects in the present investigation were healthy controls, and were screened using the Structured Clinical Interview for DSM IV Disorders-Non-patient edition (SCID-NP) to exclude individuals with Axis I psychiatric disorders. According to experienced psychiatrists, none of the subjects had a history of neurological disease or brain injury, evidence of significant medical illness, or an IQ lower than 70.

### Data acquisition and preprocessing

Images were obtained with a Siemens 3T Trio MRI scanner equipped with a 12-channel head coil. For each subject, we collected a 6-minute, 53-second rest scan comprising 116 contiguous echo-planar imaging (EPI) functional volumes (echo time [TE]  = 30 ms, repetition time [TR]  = 3500 ms, flip angle  = 90°, 35 slices, matrix  = 128×128, field of view [FOV]  = 240 mm, voxel size  = 1.9×1.9×3.5 mm^3^). Subjects were asked to relax with their eyes closed. A high-resolution T1-weighted magnetization-prepared rapid-gradient echo anatomical image was also acquired (TE  = 1.89 ms, TR  = 1670 ms, flip angle  = 9°, 208 slices, matrix  = 256×256, FOV  = 250 mm).

All images were preprocessed using Conn v13 (http://www.nitrc.org/projects/conn/) [24] and SPM8 (http://www.fil.ion.ucl.ac.uk/spm). Preprocessing was performed as follows. After discarding the first four scans to allow for signal stabilization, each subject's rs-fMRI data were corrected for slice-timing, realigned to their first scan, spatially normalized to the MNI template in SPM8, resampled to 3×3×3 mm^3^ and 4×4×4 mm^3^, and spatially smoothed with a 6-mm FWHM Gaussian kernel. During fMRI, all participants included in this study exhibited (i) spatial movement less than 1.5 mm in any direction or 1.5° in any rotation and (ii) volume-level mean framewise displacement (FD) <0.30 (mean FD across all subjects, 0.12±0.05) [25]. Further preprocessing of rs-fMRI data was performed using the Conn v13 toolbox. Data were despiked. As recommended by a recent study [26] we then applied a simultaneous band-pass filtering (0.009–0.1 Hz) and nuisance regression approach, which provides better control of nuisance variability to reduce the impact of physiological and other non-neural artifacts on connectivity analyses. Nuisance variables included six motion parameters and their first derivatives, five principal components each from white matter and cerebrospinal fluid masks (obtained using a component-based noise correction method, CompCor [27]), and a linear detrending term (a total of 23 regressors).

### Functional connectivity-based parcellation

We used a method (i.e., *K*-means clustering analysis on the average connectivity matrix across subjects) similar to that used by Deen et al. [20] and Kahnt et al. [21] to parcellate the caudate and putamen, respectively, in terms of resting-state FC (RSFC) profiles. The caudate and putamen were defined as ROIs according to the Automated Anatomical Labeling (AAL) atlas. All other AAL-labeled voxels (i.e., most gray matter voxels except for the striatum ROIs) and a box (−31<x<31, −32<y<9, −25<z<−3) covering the midbrain were defined as the rest of the brain based on a previous study [21]. For each subject, we initially calculated the Pearson correlation coefficient between the individual time series from the voxels in each ROI (from the 3×3×3-mm^3^ set) and those from each voxel in the rest of the brain excluding the ROIs (from the 4×4×4-mm^3^ set; downsampling was used to obtain sufficient spatial resolution for each ROI region and to meet reasonable computational and memory requirements). Then the individual correlation matrices were Fisher's *z*-transformed and averaged across subjects. To parcellate each ROI, we applied a standard *K*-means clustering algorithm implemented in MATLAB R2013a to the averaged matrix using the “correlation” option as distance measure based on a previous study [21]. For each *K* (*K* = 2–10), the algorithm was repeated 100 times with different initial centroids to select best clustering with the lowest within-cluster sums of point to cluster centroid distances.

### Stability of cluster solutions: determining the optimal number of clusters

To assess the stability of the cluster solutions we used a split-half comparison procedure to compute the variation of information (VI) metric [28], which was developed to assess the similarity (i.e., stability) of cluster assignments and was previously used to estimate the stability of FC-based parcellations [19], [21]. We randomly assigned subjects to one of two groups (*N* = 29 and *N* = 30), averaged the connectivity matrices within each group, and applied the clustering algorithm for each group and each *K*. For each value of *K*, we then compared the clustering of group 1 with the clustering of group 2 using the VI metric. The VI between two groups was defined as:
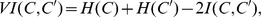
where *H*(*C*) and *H*(*C*′) are the entropies associated with clustering *C* and *C*′, respectively, and *I*(*C*, *C*′) is mutual information between the clusterings *C* and *C*′ (i.e., the information that one clustering has about the other). *H*(*C*) and *I*(*C*, *C*′) were calculated as follows:
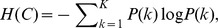
and
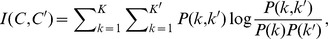



*P*(*k*) is the probability that a voxel belongs to cluster *C_k_*. *P*(*k*, *k*′) is the probability that a voxel belongs to *C_k_* in clustering *C* and to *C′_k′_* in clustering *C′*. *P*(*k*) and *P*(*k*, *k*′) were calculated as follows:
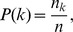
and
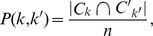
where *n_k_* is the number of voxels in cluster *C_k_*, and *n* is the total number of voxels in the ROI mask (i.e., caudate or putamen). This procedure was repeated 100 times, each time generating new random split-half groups. High VI values indicate low similarity (low stability) between clusterings of the two groups, whereas low VI values indicate high similarity (high stability). Thus, we determined the optimal *K* by computing the mean VI across the 100 permuted groups for each *K* and selecting the smallest *K* solution (i.e., most parsimonious and stable) for which the corresponding VI was statistically indistinguishable from that of the *K* – 1 solution like previous studies [19], [21]. Further details of the stability analysis can be found elsewhere [21], [28].

### Functional connectivity of striatal subdivisions based on the whole-brain mask

To examine seed-to-voxel FC for striatal subdivisions identified with the clustering analysis, a multiple regression analysis using the general linear model (GLM) implemented in SPM8 was performed for each subject including the averaged time series from each cluster as regressors [10], [21]. For example, for the *K* = 2 cluster solution, the average time series from all voxels belonging to cluster 1 and those from cluster 2 were entered simultaneously in the GLM. In this way, the parameter estimates associated with each regressor (i.e., the averaged time series from cluster 1 as a seed mask) reflected the unique variance of the latter after fitting all other regressors (cluster 2). Single-subject contrast maps for each cluster were used in a random-effect one-sample *t*-test to identify significant positive and negative connectivity at the group level. We report all results by thresholding individual voxels at *p*<0.001, uncorrected, and then applying a topological cluster-size family-wise error (FWE) of *p*<0.05 to control for multiple comparisons across the whole brain [29]. We further investigated the FC between striatal subdivisions and all other brain regions segmented based on the AAL atlas using the ROI-to-ROI approach.

### Functional connectivity of striatal subdivisions based on the CSTC mask

Based on previous research [30], [31], the following brain regions belonging to the CSTC loops were used to create the CSTC mask: cortical areas including the primary somatosensory areas (BA 1, 2, 3), primary motor area (BA 4), SMA (BA 6), frontal eye field (FEF, BA 8), DLPFC (BA 9, 46), medial OFC (med-OFC, BA 11), lateral OFC (BA 47), ventrolateral prefrontal cortex (VLPFC, BA 44, 45), anterior cingulate cortex (ACC, BA 24, 32), posterior parietal cortex (BA 5, 7, 39, 40), inferior temporal cortex (BA 20), and superior temporal cortex (BA 22); subcortical regions including the caudate, putamen, pallidum, thalamus, hippocampus, and amygdala. Thus, to improve functional specificity, a total of 23 subcortical regions were defined based on the AAL atlas, and cortical areas were defined based on the BA atlas [32], [33]. We subdivided the striatum as well as the thalamus based on its FC with other brain regions in the CSTC mask because it is known that the CSTC loops are organized into parallel pathways linking distinct cortical areas with specific striatal and thalamic nuclei [1], [3]. We then calculated the FC between the segmented cluster regions for each striatal and thalamic region and for other brain regions in the CSTC mask using the ROI-to-ROI approach.

## Results

### Stability of cluster solutions: the optimal *K*

**Figure 1:**
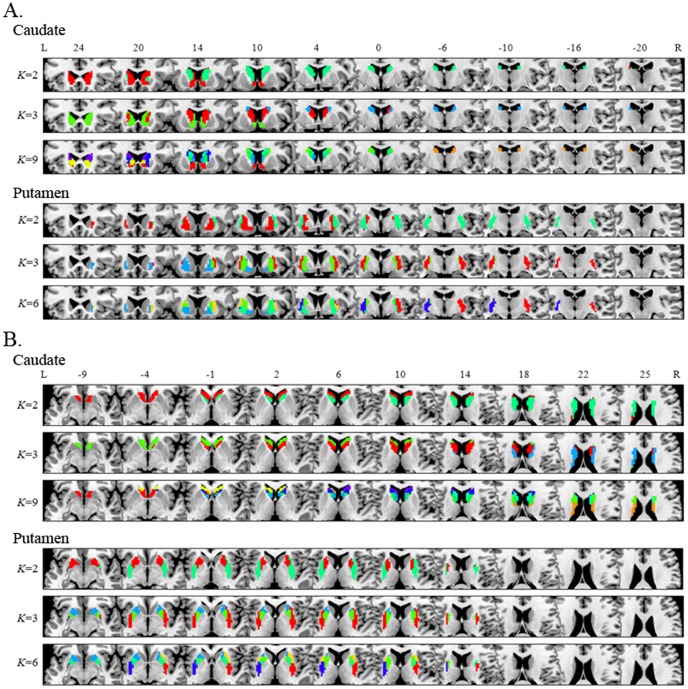
Functional connectivity-based parcellation of the caudate (A) and putamen (B) for cluster solutions with different *K*. Part A: Coronal view showing the caudate clusters identified by *K* = 2, 3, and 9 and the putamen clusters identified by *K* = 2, 3, and 6. Part B: Axial view for these same results. With an increase in *K*, the functional subdivisions of the caudate and putamen were much more detailed and segmented along the ventro-dorsal, anterior-posterior, or medio-lateral axis. All parcellation results (i.e., *K* = 2–10) are presented in Figure S1 and S2.

**Figure 2:**
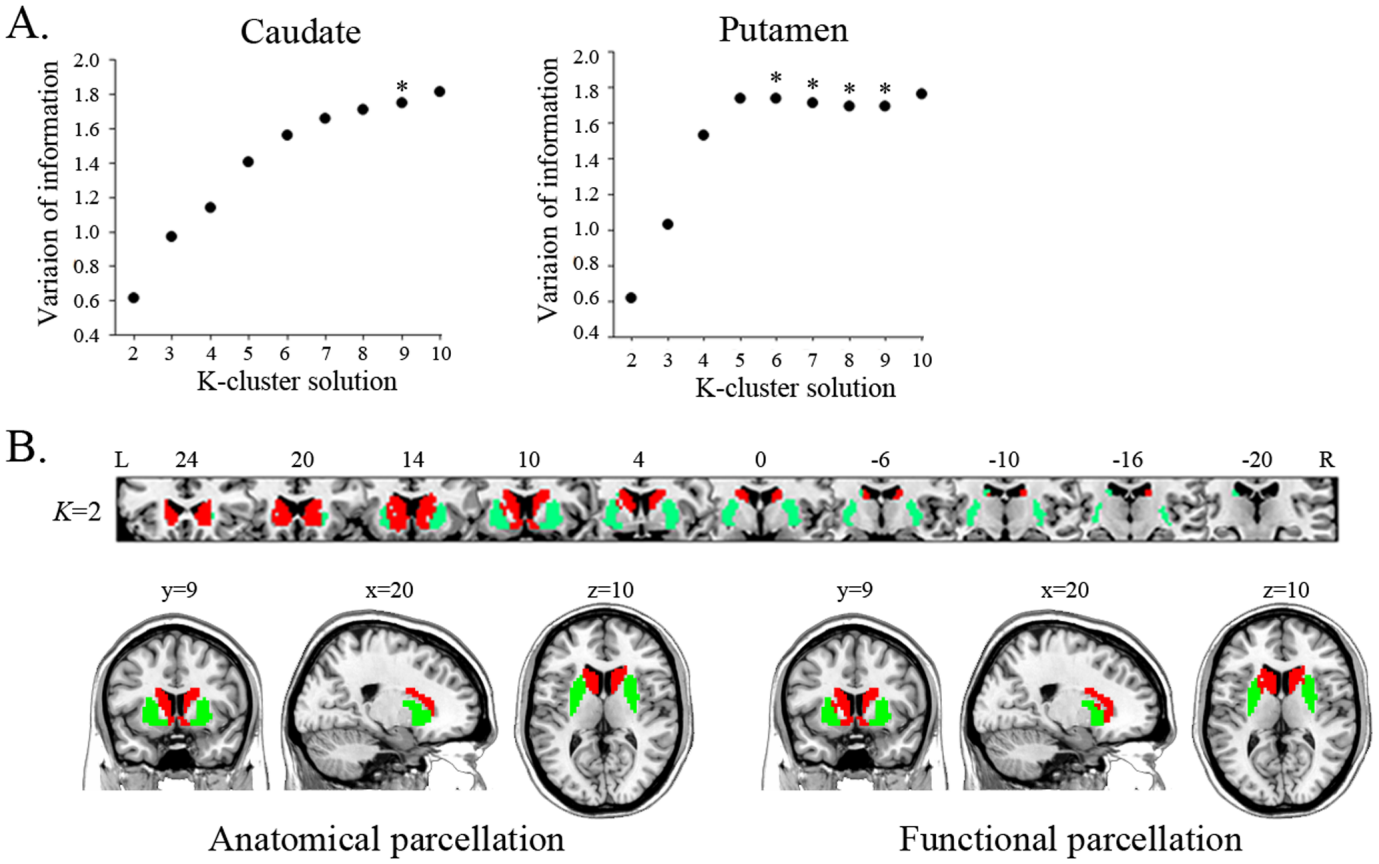
Stability of cluster solutions for the caudate and putamen and functional parcellation of the entire striatum mask. Part A: VI scores for the caudate and putamen, respectively, based on a split-half comparison procedure with permuted groups. The graph plots the mean VI across 100 permutations for each *K* cluster solution. The asterisks indicate the number of subdivisions that can be stably estimated by the clustering algorithm, in which VI is statistically indistinguishable from that of the *K*–1 solution. There were no VI differences between *K* = 8 and 9 (t = −1.76, p = 0.08) for the caudate, and no differences between *K* = 5 and 6 (t = 0.027, p = 0.98), *K* = 6 and 7 (t = 1.04, p = 0.30), *K* = 7 and 8 (t = 0.714, p = 0.48), and *K* = 8 and 9 (t = −0.16, p = 0.87) for the putamen. Part B: Clustering result of the entire striatum mask including the caudate and putamen in the *K* = 2 cluster solution. The pattern of functional subdivision (right panel) in the entire striatum is very similar to its anatomical subdivision (left panel).

We applied the *K*-means clustering algorithm to examine the functional subdivisions of the striatum. Although this analysis was performed for *K* = 2–10 for the caudate and putamen (Fig. S1 and S2), we focused particularly on specific numbers of subdivisions (Fig. 1): *K* = 2 for both the caudate and putamen at the simplest level; *K* = 3 for both the caudate and putamen based on previous DTI tractography-based parcellation studies [5], [6]; and *K* = 9 and 6 for the caudate and putamen, respectively, according to the number identified by the VI. Consistent with previous studies [19], [21], the stability of cluster solutions across the 100 permuted groups decreased (VI increased) as *K* increased (Fig. 2A). Whereas only the VIs from the *K* = 8 and 9 cluster solutions for the caudate were not statistically significant, differences between the VIs from several *K* and *K* – 1 (i.e., *K* = 5 and 6, *K* = 6 and 7, *K* = 7 and 8, and *K* = 8 and 9) in the putamen were statistically indistinguishable. Accordingly, the *K* = 9 and 6 cluster solutions for the caudate and putamen, respectively, are optimal when they are the smallest *K*s for which the VI does not significantly increase compared with the *K* – 1.

### Connectivity-based parcellation of the striatum

**Figure 3:**
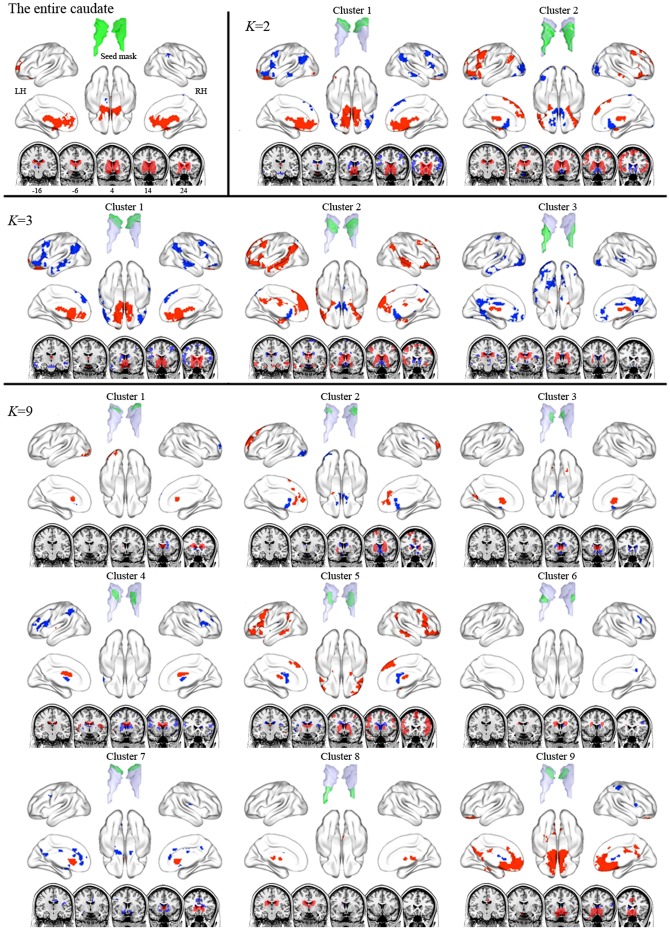
Functional connectivity maps of the entire caudate and its subdivisions in the *K* = 2, 3, and 9 cluster solutions. Red and blue indicate areas showing positive functional correlation and negative functional correlation with seed region, respectively. The ventral and dorsal caudate clusters were positively connected to areas involved in emotional processes and cognitive processes, respectively.

In the caudate (Fig. 1 and Fig. 3), the *K* = 2 cluster solution revealed that cluster 1 covers the anterior–ventral surface, which is consistent with the head of caudate, and cluster 2 covers the middle and posterior surface of the caudate. Although some voxels in cluster 1 were isolated from the main section, they were very rare and blended into neighbors according to the higher *K*; we excluded these isolated voxels in the seed mask for cluster 1 when performing subsequent FC analyses with striatal subdivisions. The *K* = 3 cluster solution also revealed the same anterior–ventral cluster (cluster 1), and the other cluster covering the middle and posterior surface in the *K* = 2 cluster solution was divided along the anterior–posterior axis into the middle cluster (cluster 2) and posterior–dorsal cluster (cluster 3). The spatial pattern of these three clusters corresponds to the head, body, and tail of the caudate. According to the *K* = 9 cluster solution, clusters 1, 2, and 3 in the *K* = 3 cluster solution were divided into three (cluster 1, 7, and 9), four (cluster 2, 3, 4, and 5), and two (cluster 6 and 8) compartments, respectively.

**Figure 4:**
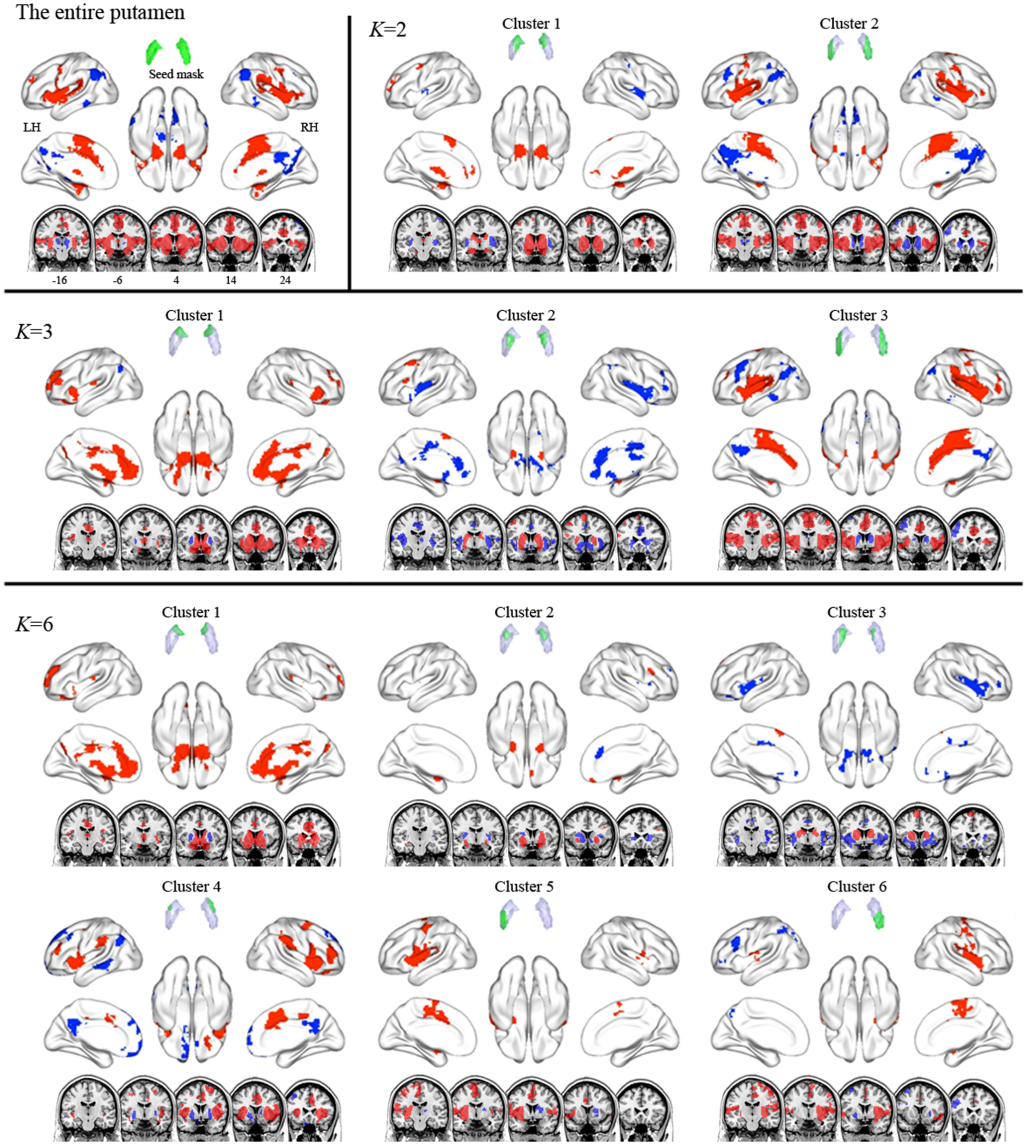
Functional connectivity maps of the entire putamen and its subdivisions in the *K* = 2, 3, and 6 cluster solutions. Red and blue indicate positive and negative functional correlation with the seed region, respectively. The rostral putamen clusters were positively connected to areas involved in emotional and cognitive processes, while the caudal putamen clusters were positively connected to motor areas.

In the putamen (Fig. 1 and Fig. 4), the *K* = 2 cluster solution revealed that the putamen divides along the medial–lateral axis, with cluster 1 covering the lateral surface and cluster 2 covering the medial surface. According to the *K* = 3 cluster solution, the cluster 1 in the *K* = 2 cluster solution split along the anterior–posterior axis (clusters 1 and 2), and the lateral cluster (cluster 3) was conserved. In the *K* = 6 cluster solution, clusters 2 and 3 in the *K* = 3 cluster solution divided into two (cluster 2 and 3) and three parts (cluster 4, 5, and 6), respectively.

We also conducted an exploratory clustering analysis of the entire striatum mask, including both the caudate and putamen, to investigate whether the functional subdivision of the striatum based on RSFC is related to its anatomical structure. The results of this analysis revealed that the functional segmentation between the caudate and putamen in the striatum largely corresponded to the anatomical segmentation between these regions by the internal capsule, showing a correspondence rate of 92% (Fig. 2B).

### Whole-brain connectivity of striatal subdivisions

We performed seed-to-voxel FC analyses with striatal subdivisions, focusing specifically on the positive connectivity maps for each cluster seed because debate about negative connectivity maps for seed regions persists [34]–[37]. The entire caudate was positively connected to medial prefrontal cortex (med-PFC), ACC, and amygdala (Fig. 3 and Table S1). In the *K* = 2 cluster solution, cluster 1 was positively connected to med-PFC and ACC, which is similar to the FC map of the entire caudate, whereas cluster 2 was positively linked to DLPFC, lat-OFC, superior med-PFC, amygdala, IPC, and MTC. Interestingly, the positive connectivity map of cluster 2 was similar to the negative connectivity map of cluster 1, suggesting an anti-correlation between the two clusters. In the *K* = 3 cluster solution, the positive connectivity maps of cluster 1 and cluster 2 showed patterns similar to those of the *K* = 2 cluster solution, but extended to more distributed regions. Cluster 3 showed positive connectivity with thalamus and amygdala. In the *K* = 9 cluster solution, the positive connectivity maps of clusters 9 and 5 showed patterns similar to those of clusters 1 and 2 in the *K* = 3 cluster solution, although these extended to more distributed regions.

The entire putamen was positively connected to precentral cortex, SMA, insula, DLPFC, amygdala, and STC (Fig. 4 and Table S2). In the *K* = 2 cluster solution, cluster 1 showed positive connectivity with DLPFC, med-PFC, ACC, and SMA, whereas cluster 2 exhibited positive connectivity with precentral cortex, SMA, amygdala, and insula. According to the *K* = 3 cluster solution, clusters 1 and 2 respectively showed positive connectivity with DLPFC, med-PFC, ACC, cuneus, precuneus, amygdala, insula, and STC; and with DLPFC, SMA, and amygdala. The positive FC pattern of cluster 3 was similar to that of cluster 2 in the *K* = 2 cluster solution. In the *K* = 6 cluster solution, the positive connectivity map of cluster 3 seems to divide into that of clusters 4, 5, and 6.

**Figure 5:**
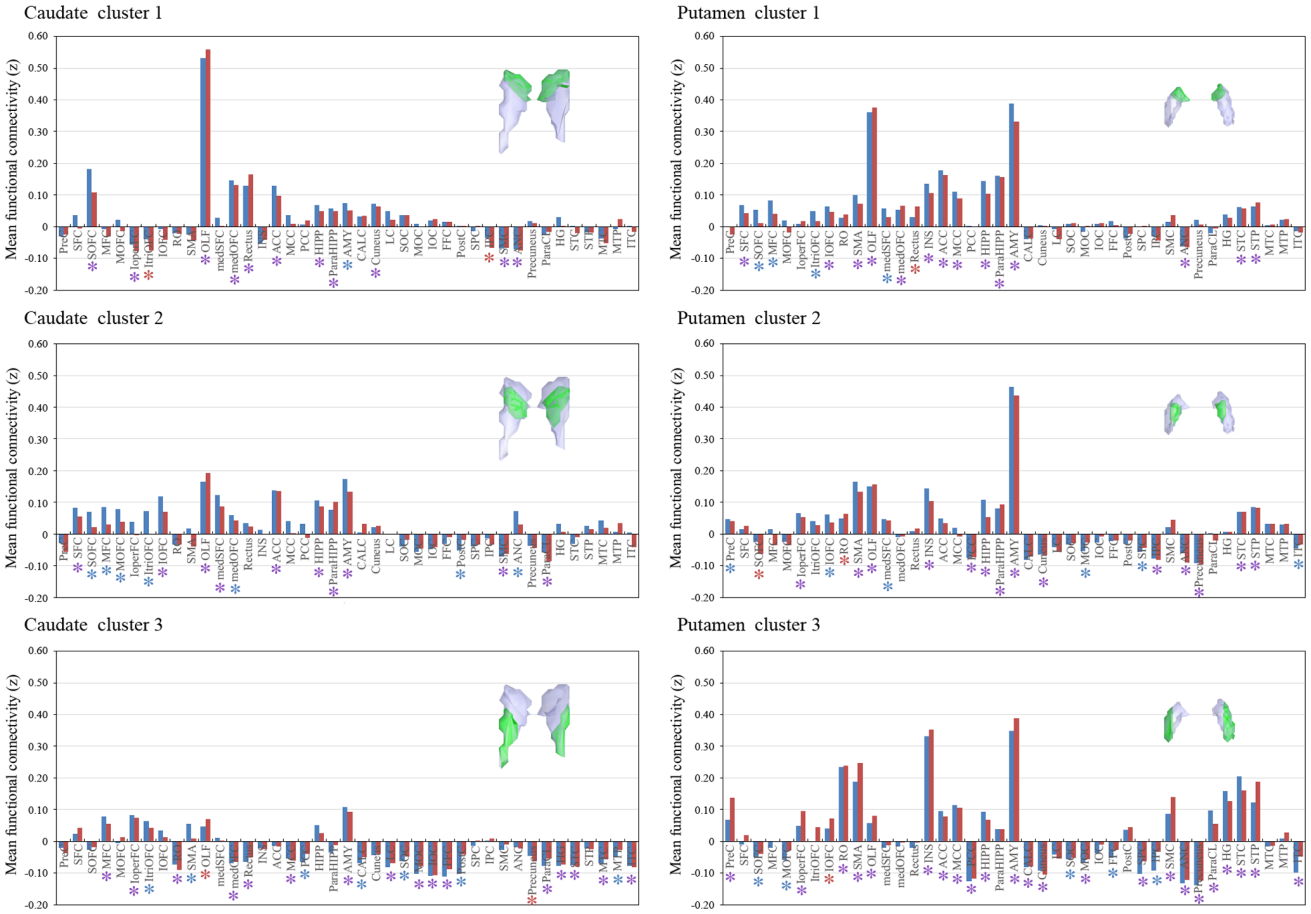
ROI analysis results for functional connectivity between striatal clusters identified using clustering algorithms (*K* = 3) and other brain regions, segmented based on the AAL atlas. The x-axis indicates brain regions, and the y-axis indicates the strength of functional connectivity between each cluster as a seed region and other brain regions, as correlation z scores. The red and blue bars respectively indicate the area located in the right and left hemisphere. The red, blue, and purple asterisks respectively indicate that these regions located on the right, left, or bilaterally had significant functional connectivity with the cluster region under a false discovery rate threshold of q<0.05. All ROI-to-ROI results for striatal clusters identified by different *K* are presented in Figure S3–S5.

ROI-to-ROI analyses revealed specific connections between functional striatal subregions and specific cortical regions segmented by the AAL atlas (Fig. 5). For example, in the *K* = *3* cluster solution (Fig. 5), caudate cluster 1 and 2 showed strong positive connectivity with ventral med-PFC (i.e., olfactory cortex (OLF) and med-OFC) and superior med-OFC and moderate connectivity with frontal cortical regions and amygdala. The putamen's cluster 1 exhibited strong positive connectivity with limbic areas including the OLF, amygdala, and ACC, whereas cluster 3 showed strong positive connectivity not only with limbic areas including the OLF, amygdala, and insula but also with motor areas including SMA. We provide the results of ROI-to-ROI analyses for the caudate and putamen subdivision defined by different *K* in Fig. S3–S5.

### ROI-to-ROI connectivity between CSTC regions

**Figure 6:**
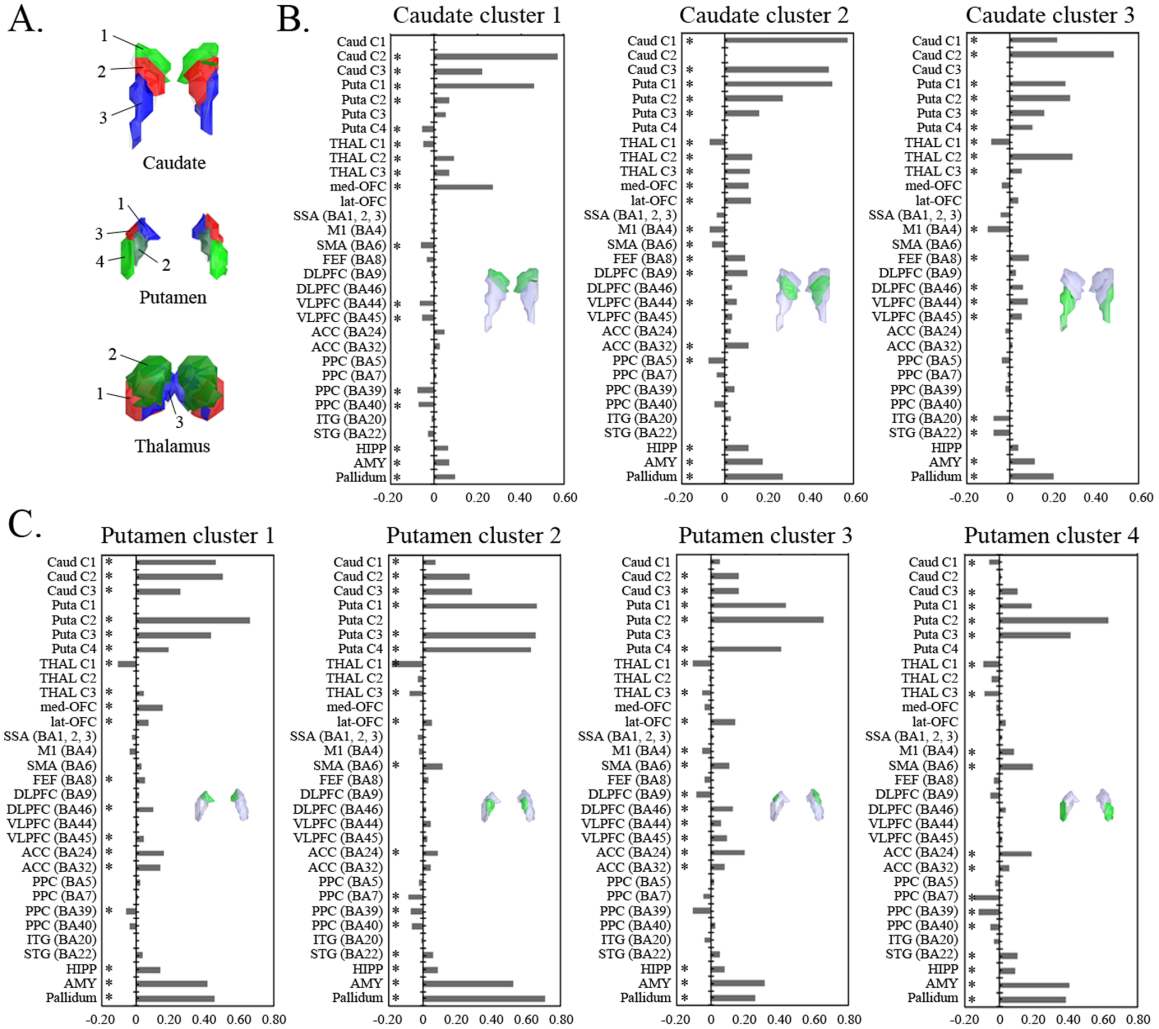
ROI analysis results for functional connectivity between striatal and thalamic clusters and other regions that belong to CSTC loops. Part A: Clustering results of the caudate, putamen, and thalamus based on functional connectivity patterns across regions belonging to CSTC loops. Part B: Correlations of the caudate clusters with other regions. Part C: Correlations of the putamen clusters with other regions. The x-axis indicates the strength of functional connectivity between the clusters and other brain regions as correlation z scores, and the y-axis indicates brain regions. For simplicity, we focused on results from bilateral ROI regions. The black asterisks indicate regions with significant functional connectivity with the cluster regions under a false discovery rate threshold of q<0.05.

We performed *K*-means clustering analysis across subjects based on the FC between regions belonging to the CSTC mask and then determined the optimal *K* using the VIs mentioned above (*K* = 3, 4, and 3 for the caudate, putamen, and thalamus, respectively). The result of the *K* = 3 cluster solution for the caudate was described above (Fig. 6A). The putamen in the *K* = 4 cluster solution split along the medial–lateral and anterior–posterior axes into anterior–medial (cluster 1), posterior–medial (cluster 2), anterior–lateral (cluster 3), and posterior–lateral clusters (cluster 4) (Fig. 6A, see Fig. S6 for results from seed-to-voxel FC analyses with putamen subregions in the *K* = 4 cluster solution). The thalamus in the *K* = 3 cluster solution (Fig. 6A) split into the lateral–ventral (cluster 1), lateral–dorsal (cluster 2), and medial–dorsal (cluster 3) clusters. We calculated FC between segmented cluster regions for the striatum and thalamus and other brain regions in the CSTC mask using an ROI-to-ROI approach (Fig. 6B and 6C).

We found that certain striatal subregions had stronger positive connections with some cortical regions than with others. The anterior part of putamen (clusters 1 and 3) exhibited positive connectivity with frontal areas, whereas the posterior part of the putamen (clusters 2 and 4) showed positive connectivity with motor areas including SMA. In addition, all putamen clusters showed stronger positive connectivity with the amygdala than other regions, except striatal regions. We also found positive connectivity between striatal subregions and specific thalamic clusters. Clusters 1, 2, and 3 of the caudate exhibited positive connectivity with those of the putamen, whereas cluster 4 of the putamen showed positive connectivity with only the caudate's cluster 3. All caudate clusters showed positive connectivity with thalamus clusters 2 and 3 as well as negative connectivity with thalamus cluster 1. Cluster 1 of the putamen showed positive connectivity with thalamus cluster 3. All putamen clusters showed negative connectivity with thalamus cluster 1. We also provide these results rearranged in order of the strength of FC between pairs of regions (Fig. S7).

## Discussion

**Figure 7:**
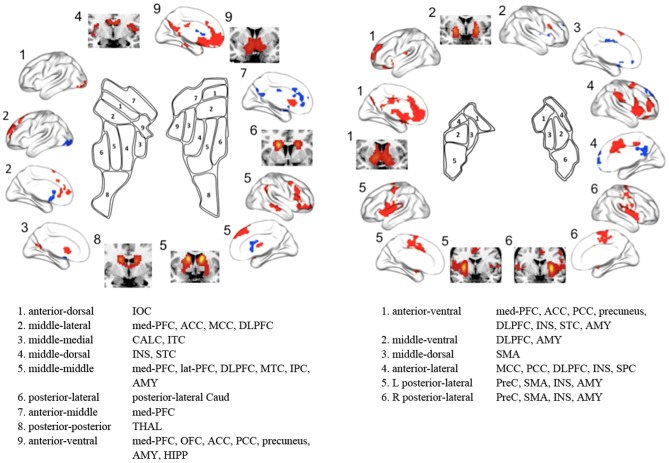
Summary of striatal subdivisions and their functional connectivity maps. The schematic illustrates parcellations based on the optimal *K* (9 and 6 for the caudate and the putamen respectively). Regions showing significant functional connectivity to each striatal cluster are depicted on the surface and the section of the brain, and are also listed. IOC, inferior occipital cortex; med-PFC, medial prefrontal cortex; ACC, anterior cingulate cortex; MCC, middle cingulate cortex; DLPFC, dorsolateral prefrontal cortex; CALC, calcarine cortex; ITC, inferior temporal cortex; INS, insula; STC, superior temporal cortex; lat-PFC, lateral prefrontal cortex; MTC, middle temporal cortex; IPC, inferior parietal cortex; AMY, amygdala; Caud, caudate; THAL, thalamus; OFC, orbitofrontal cortex; PCC, posterior cingulate cortex; HIPP, hippocampus; SMA, supplementary motor area; SPC, superior parietal cortex; PreC, precentral cortex.

In the present study, we not only characterized the functional subdivisions of the human striatum using rs-fMRI and unsupervised clustering algorithms without any specific reference (i.e., anatomical or functional targets) but also delineated distinct FC maps for striatal subdivisions. Our results are in agreement with recent *in vivo* anatomical connectivity studies [6], [9] as well as with contemporary models of multiple parallel CSTC loops [3], [38] showing connections between specific striatal subdivisions and different cortical areas according to associated cognitive, affective, and motor functions (i.e., increased FC between the dorsal and ventral striatum and cognitive and affective control areas, respectively, as well as between the dorsolateral putamen and motor areas) (Fig. 7). Our findings demonstrate that FC patterns during the resting-state can be a reliable feature in parcellating the striatum and in the *in vivo* identification of distinct functional networks of CSTC loops.

Consistent with the anatomical division of the striatum by the internal capsule, we confirmed intrinsic functional distinctions between the caudate and putamen and further functional subdivisions within each of these structures. Although prior models of CSTC loops [1], [39] and previous imaging studies [40] suggested that the affective and cognitive loops involve the caudate but not the putamen and that the motor loop involves the putamen more than it involves the caudate, more recent studies have demonstrated that the putamen is also associated with various cognitive processes, such as learning and memory for stimulus–response associations [41], [42]. Our FC results regarding striatal subdivisions were consistent with this suggestion and with previous striatal parcellation results [12], as the caudate clusters were positively connected to areas involved in affective and motivational processes, such as the med-PFC and OFC, and areas involved in executive cognitive processes, such as the DLPFC, lat-OFC, IPC, and MTC. However, the putamen clusters were positively connected to motor areas, such as precentral cortex and SMA, as well as to cognitive control areas, such as DPFC, med-PFC, ACC, insula, and STC. Specifically, in terms of rostral/caudal (anterior/posterior) gradients, the rostral putamen clusters were positively connected to affective and cognitive control areas, whereas the caudal putamen clusters were positively connected to more motor control areas. In terms of dorsal/ventral gradients, the ventral striatal clusters, particularly the ventromedial part of the striatum, were positively connected to areas involved in affective and motivational processes, whereas the dorsal striatal clusters were positively connected to cognitive/executive areas. Moreover, dorsal putamen clusters, particularly the dorsolateral parts of the putamen, were positively connected to motor areas. We found that the patterns of functional subdivision in the striatum and their FC were highly consistent with current accounts of functional distinctions in the striatum (i.e., dorsolateral/ventromedial functional organization) [43], [44] and functional co-activation patterns based on a meta-analysis of 126 task-based functional studies [16]. Our findings are also consistent with a recent FC study with seeds defined based on these co-activation patterns [10].

The present striatal parcellations are strikingly similar to those of Choi et al. [12] except that we do not observe clusters located commonly in both the caudate and putamen, likely because of differences in the striatal ROIs used in each study (discussed below as one of the limitations). Choi et al. [12] reported functional coupling between specific striatal subdivisions and cortical networks by assigning each voxel in the striatum to its most correlated network among cognitive, affective, motor networks (i.e., the default mode, fronto-parietal, ventral attention, dorsal attention, visual, limbic, and motor networks) identified by Yeo et al. [23]. For example, the motor and fronto-parietal networks were functionally linked to posterior putamen (the putamen clusters 5 and 6 in this study) and dorsal caudate (the caudate cluster 5) respectively. They found that the motor, limbic, default mode, fronto-parietal, and ventral attention networks were robustly represented in the striatum, while the dorsal attention and visual networks were nearly not represented. Consistent with these findings, we observed that the patterns of FC maps to the striatal subdivisions were similar to the motor (putamen clusters 5 and 6), limbic (putamen cluster 1 and caudate clusters 7 and 9), ventral attention (putamen cluster 4), fronto-parietal (caudate cluster 5), and default mode networks (caudate clusters 2, 3, and 9, and putamen cluster 1) (Fig. 7). In addition, we showed that parcellation patterns from the whole-brain mask and the CSTC mask were very similar. Therefore, the present study confirms previous findings based on RSFC profiles, showing similarity between the striatal parcellation patterns obtained by the whole brain mask and those obtained by defined targets (i.e., cerebral networks and CSTC mask), and contributes additional evidence that specific striatal zones are functionally linked to distributed cerebral networks involved in specific functions, rather than discrete portions of a specific lobe or cortical region [12].

The pattern of striatal subdivisions became more fine-grained as the number (*K*) of cluster solutions increased. Interestingly, functional subdivisions (i.e., the split from *K* – 1 to *K* clusters) appeared to be almost hierarchical, although the *K*-means clustering algorithm is a non-hierarchical method. For example, cluster 2 of the caudate in the *K* = 2 cluster solution divided into clusters 2 and 3 in the *K* = 3 cluster solution, and cluster 2 in the *K* = 3 cluster solution further divided into clusters 2, 3, 4, and 5 in the *K* = 9 cluster solution. Such a pattern was also observed in the putamen. Furthermore, with the split from *K* – 1 to *K* clusters, the spatial pattern of the FC between cortical regions and striatal clusters became more detailed and segmented. Previous studies have reported a hierarchically clustered organization in functional brain networks [45] and in functional subdivisions of cortical areas [21]. Further work using hierarchical clustering algorithms should clarify the issue of hierarchical organization in the striatum.

We observed patterns of anticorrelation between the FC maps associated with different striatal subdivisions. Specifically, the positive connectivity map of caudate cluster 2 in the *K* = 2 cluster solution was spatially similar to the negative connectivity map of cluster 1 and vice versa. Many previous studies have demonstrated two opposing brain networks both during task performance and at rest: the task-negative network (also known as the default mode network) and its anticorrelated network (also called the task-positive network or attention control network) [46], [47]. The default mode network is thought to be related to emotional and self-referential processing and includes cortical midline structures including the med-PFC and PCC, whereas the attention control network is known to be related to executive cognitive processing and includes DLPFC and posterior parietal cortex. Consistent with this notion, FC maps involving the ventral caudate and the dorsal caudate are similar to the default mode network and attention control network, respectively. Several previous studies also reported an anticorrelation between inferior ventral striatum seeds and areas involved in cognitive and/or motor control [10], [33].

Similar to findings from seed-based FC analyses, the results of the ROI-to-ROI analyses based on both the AAL and the BA atlases support the existence of multiple parallel CSTC loops linking the striatum to cortical regions. These data provide evidence for distinct connectivity of ventral and dorsal striatum with affective and cognitive processing areas, respectively, and of rostral and caudal putamen with cognitive and motor processing areas, respectively. Based on contemporary CSTC models, it is suggested that although various cognitive processes relate to the ventral anterior and dorsomedial thalamus, motor functioning relates to the ventrolateral thalamus [1]. Consistent with this notion, cluster 2 (dorsal part) and cluster 3 (medial part) of the thalamus were positively connected with all caudate clusters and with different cortical regions associated with affective and cognitive processes, such as the medial and lateral frontal cortices. However, cluster 1 (ventrolateral part) was negatively connected to all striatal clusters. We also found stronger FC between putamen clusters and the amygdala than other cortical regions. This finding is consistent with previous studies reporting structural [7], [48] and functional connections [49] between the putamen and amygdala. Recent studies have suggested that the putamen and amygdala are part of the salience network, as increased activity has been shown in these regions in response to salient stimuli, such as nociceptive [48], [50] or appetitive stimuli [51]. However, the strong correlation between those regions may also be due to the impact of signal bleeding between proximal structures as we discuss below.

Several potential limitations need to be considered when interpreting our findings. First, although our results are consistent with previous studies of anatomical connectivity, the precise relationship between functional and anatomical connections in the CSTC loops remains unclear. Further studies combining DTI and fMRI techniques are needed to improve our understanding of the *in vivo* relationship between anatomy and function in these loops. Second, our findings were based on rs-fMRI rather than on task-based fMRI. Recent studies have demonstrated that functional networks are modulated by cognitive and affective states [33], [52]. Thus, additional studies are needed to investigate whether the pattern of functional organization in the striatum changes or is preserved during task performance. Third, we tried to remove confounding factors that could cause motion-related effects and spurious negative correlations in seed-based FC analysis; we censored motion using a strict criterion and applied a CompCor strategy [27]. This approach allows interpretation of anticorrelations, as there was no global signal regression for removal of physiological and other confounds. However, debate about negative connectivity maps for seed-based FC analysis still persists [36], [37]. Fourth, there may be an impact of signal bleeding due to proximity, particularly in FC of the insula and amygdala to the putamen. Previous studies reported such effect on the parcellation pattern based on RSFC and tried to eliminate this effect by regressing out the mean signal of adjacent regions [12], [22], [23]. Choi et al. [12] showed that the removal of the insula signal caused the posterior putamen assignment to switch from the ventral attention network located adjacently in the insula to the motor network, which agrees with monkey anatomical tact-tracing studies [53]. Additionally, anatomical data show that projections from the amygdala land primarily in ventral putamen and ventral caudate [54], [55]. However, in this study the amygdala was very strongly correlated with the (more proximal) putamen, but less-strongly correlated with the caudate (e.g., Fig. 5B caudate cluster 1 versus putamen clusters 1 and 2). Thus in ROI-to-ROI analysis, the strong correlation of putamen to the insula or amygdala (i.e., Fig. 5 and 6) may be a result of signal bleeding due to proximity between them. However, our voxel-based FC analysis, in which signals from all clusters in a specific cluster solution were modeled simultaneously using multiple regression, showed a strong correlation between the posterior putamen and motor areas and between the ventral caudate and amygdala. For this reason, the prior study by Choi et al. [12] was least certain about the details of results in the putamen, particularly with respect to the ROI-to-ROI result. We also focused on the more certain caudate results and voxel-based FC results. Fifth, the issue of proper imaging duration to perform the stable FC estimates may be of concern. Based on Van Dijk et al.'s study that showed stable estimates of FC strength with 5 minutes of data acquisition [56], researchers in many current rs-fMRI studies have used data obtained for as little as 5–7 minutes. However, in contrast to this previous result, more recent studies have reported improvements in test-retest reliability and across-session similarity of FC estimates by increasing the scan duration to greater than 10 minutes (recommended durations include 15–25 minutes or longer by Anderson et al. [57] and 9–13 minutes or longer by Birn et al. [58]). However, increasing the scan duration can result in the increase of head motion, which affects RSFC. Finally, despite being physically separated by the internal capsule, the caudate and putamen share similar cell types and are considered as a single complex, the striatum, which are all part of one nucleus continuous at their bases [59], [60]. In other words, the striatum is a highly integrative structure without sharp borders, in contrast to the sharp borders imposed by the winner-take-all clustering solutions. In addition, based on the results of the prior study by Choi et al. [12], it is conceivable that some individual clusters may encompass portions of both the caudate and putamen. In this regard, our parcellation strategy (i.e, to perform the clustering analysis separately on the caudate and putamen as two independent structures) may be inappropriate to address integration within the striatum and across parallel CSTC loops.

In summary, we confirmed prior results [12] showing functional coupling between specific striatal subregions and cerebral networks by using rs-fMRI and an unsupervised clustering algorithm without specific target references. In addition, the present study examined the intrinsic functional organization in the caudate and putamen according to different cluster solutions. The functional subdivisions identified are consistent with the striatal subdivisions based on anatomical connectivity as well as with the dorsal/ventral and rostral/caudal functional distinctions in the striatum. Moreover, the distinct connectivity patterns in these striatal clusters were consistent with contemporary models of multiple parallel CSTC loops, with specific clusters showing connectivity with cortical areas involved in cognitive, affective, or motor processes. The current method improves our understanding of the contributions of striatal subregions to the performance of specific cognitive, affective, and motor tasks, both in rodents (which lack sharp boundaries in the striatum) and humans. Our findings are relevant to understanding disorders characterized by atypical striatal function, and may also be useful for preoperative neurosurgical mapping.

## Supporting Information

Figure S1 **Coronal view showing functional connectivity-based parcellation of the caudate (A) and putamen (B) for cluster solutions with different** ***K*** **(2–10).** With an increase in *K*, the functional subdivisions of the caudate and putamen were much more detailed and segmented along the ventro-dorsal, anterior-posterior, or medio-lateral axis.
(PDF)

Figure S2 **Axial views showing functional connectivity-based parcellation of the caudate (A) and putamen (B) according to different** ***K*** **(2–10) cluster solutions.** With an increase in *K*, the functional subdivisions of the caudate and putamen were much more detailed and segmented.
(PDF)

Figure S3 **ROI analysis results for functional connectivity between striatal clusters identified by using clustering algorithms (** ***K*** **= 2) and other brain regions segmented based on the AAL atlas.** Part A: The FC between the entire voxels within the caudate and putamen respectively and other regions. Part B: The FC between clusters identified by *K* = 2 and other regions (B). The x-axis indicates brain regions, and the y-axis indicates the strength of functional connectivity between each cluster as a seed region and other brain regions, as correlation z scores. The red and blue bars respectively indicate the area located in the right and left hemisphere. The red, blue, and purple asterisks respectively indicate that these regions located in the right, left, and bilateral hemisphere had significant functional connectivity with the cluster region under a false discovery rate threshold of q<0.05.
(PDF)

Figure S4 **ROI analysis results for functional connectivity between caudate clusters identified by** ***K*** **= 9 cluster solution and other brain regions, segmented based on the AAL atlas.** The x-axis indicates brain regions, and the y-axis indicates the strength of functional connectivity between each cluster as a seed region and other brain regions, as correlation z scores. The red and blue bars respectively indicate the area located in the right and left hemisphere. The red, blue, and purple asterisks respectively indicate that these regions located in the right, left, and bilateral hemisphere had significant functional connectivity with the cluster region under a false discovery rate threshold of q<0.05.
(PDF)

Figure S5 **ROI analysis results for functional connectivity between putamen clusters identified by** ***K*** **= 6 cluster solution and other brain regions, segmented based on the AAL atlas.** The x-axis indicates brain regions, and the y-axis indicates the strength of functional connectivity between each cluster as a seed region and other brain regions, as correlation z scores. The red and blue bars respectively indicate the area located in the right and left hemisphere. The red, blue, and purple asterisks respectively indicate that these regions located in the right, left, and bilateral hemisphere had significant functional connectivity with the cluster region under a false discovery rate threshold of q<0.05.
(PDF)

Figure S6 **Functional connectivity maps of the putamen subdivisions in the** ***K*** **= 4 cluster solution.** The red and blue areas indicate those showing positive and negative functional correlation with the seed region, respectively. The rostral putamen clusters were positively connected to the areas involved in affective and cognitive processes, while the caudal putamen clusters were positively connected to motor areas.
(PDF)

Figure S7 **ROI analysis results for functional connectivity between striatal and thalamic clusters and other regions that belong to CSTC loops.** These results were rearranged in order of the strength of the FC between pairs of regions. Part A: Correlations between the caudate clusters and other regions. Part B: Correlations between the putamen clusters and other regions. The x-axis indicates the strength of functional connectivity between the cluster regions and other brain regions as correlation z scores, and the y-axis indicates brain regions, which are rearranged in order of the strength of functional connectivity. For simplicity, we focused on results from bilateral ROI regions. The black asterisks indicate that these regions had significant functional connectivity with the cluster regions under a false discovery rate threshold of q<0.05.
(PDF)

Table S1 **Peak coordinates of connectivity maps with caudate subregions.** Coordinates for peak voxels are presented in MNI space. Caud, caudate; OLF, olfactory cortex; SFC, superior frontal cortex; SMC, supramarginal cortex; IPC, inferior parietal cortex; MOFC, middle orbitofrontal cortex; MOC, middle occipital cortex; IOFC, inferior orbitofrontal cortex; MFC, middle frontal cortex; IFC, inferior frontal cortex; medSFC, medial superior frontal cortex; MTC, middle temporal cortex; HIPP, hippocampus; MTC, middle temporal cortex; ANC, angular cortex; ParaCL, paracentral lobule; SFC, superior frontal cortex; MCC, middle cingulate cortex; SMA, supplementary motor area; CALC, calcarine cortex; ACC, anterior cingulate cortex; STC, superior temporal cortex; INS, insula; IOC, inferior occipital cortex; THAL, thalamus; SOC, superior occipital cortex; PreC, precentral cortex, ITC, inferior temporal cortex, SOFC, superior orbitofrontal cortex; FFC, fusiform face cortex; AMY, amygdala; PostC, postcentral cortex; PCC, posterior cingulate cortex; medOFC, medial orbitofrontal cortex.
(PDF)

Table S2 **Peak coordinates of connectivity maps with putamen subregions.** Coordinates for peak voxels are presented in MNI space. Puta, putamen; PreC, precentral cortex; MFC, middle frontal cortex; MCC, middle cingulate cortex; LC, lingual cortex; THAL, thalamus; MOC, middle occipital cortex; ANC, angular cortex; MTC, middle temporal cortex; SMA, supplementary motor area; ACC, anterior cingulate cortex; INS, insula; PosC, postcentral cortex; STC, superior temporal cortex; IPC, inferior parietal cortex; ITC, inferior temporal cortex; IFC, inferior frontal cortex; SMC, suparmarginal cortex; MCC, middle cingulate cortex; MOC, middle occipital cortex; SFC, superior frontal cortex; SOFC, superior orbitofrontal cortex; medOFC, medial orbitofrontal cortex; SPC, superior parietal cortex; medSFC, medial superior frontal cortex.
(PDF)
