# Supplementary material for: Unravelling the Intrinsic Functional Organization of the Human Striatum: A Parcellation and Connectivity Study Based on Resting-State fMRI
Source: PLoS One. 2014 Sep 9;9(9):e106768. doi: 10.1371/journal.pone.0106768 (PMC4159235; doi:10.1371/journal.pone.0106768)
Supplement: Figure S1 — Coronal view showing functional connectivity-based parcellation of the caudate (A) and putamen (B) for cluster solutions with different K (2–10). With an increase in K, the functional subdivisions of the caudate and putamen were much more detailed and segmented along the ventro-dorsal, anterior-posterior, or medio-lateral axis. (PDF) [file pone.0106768.s001.pdf]

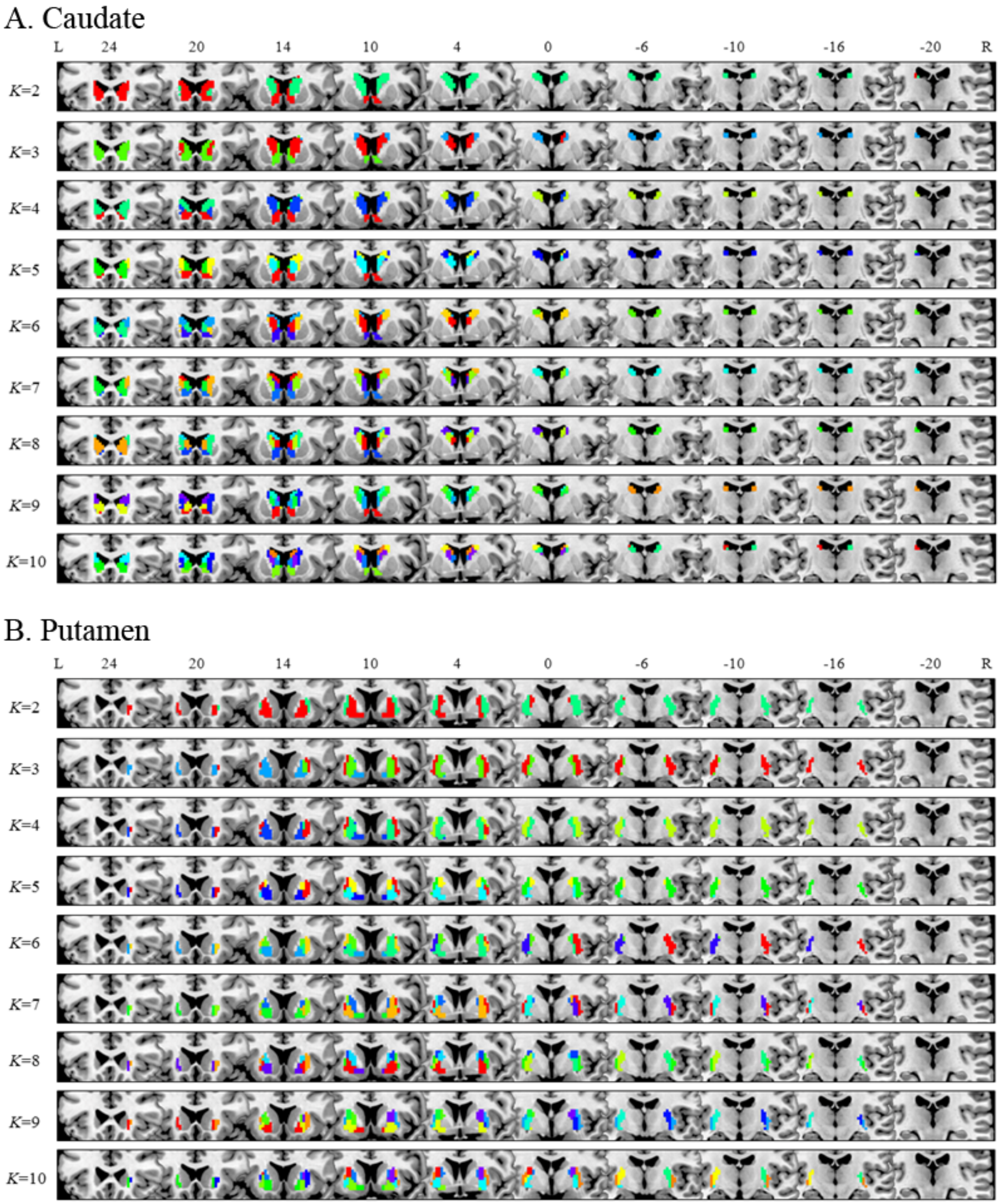

Figure S1. Coronal view showing functional connectivity-based parcellation of the caudate (A) and putamen (B) for cluster solutions with different *K* (2–10). With an increase in *K*, the functional subdivisions of the caudate and putamen were much more detailed and segmented along the ventro-dorsal, anterior-posterior, or medio-lateral axis.
