# Supplementary material for: Unravelling the Intrinsic Functional Organization of the Human Striatum: A Parcellation and Connectivity Study Based on Resting-State fMRI
Source: PLoS One. 2014 Sep 9;9(9):e106768. doi: 10.1371/journal.pone.0106768 (PMC4159235; doi:10.1371/journal.pone.0106768)
Supplement: Figure S2 — Axial views showing functional connectivity-based parcellation of the caudate (A) and putamen (B) according to different K (2–10) cluster solutions. With an increase in K, the functional subdivisions of the caudate and putamen were much more detailed and segmented. (PDF) [file pone.0106768.s002.pdf]

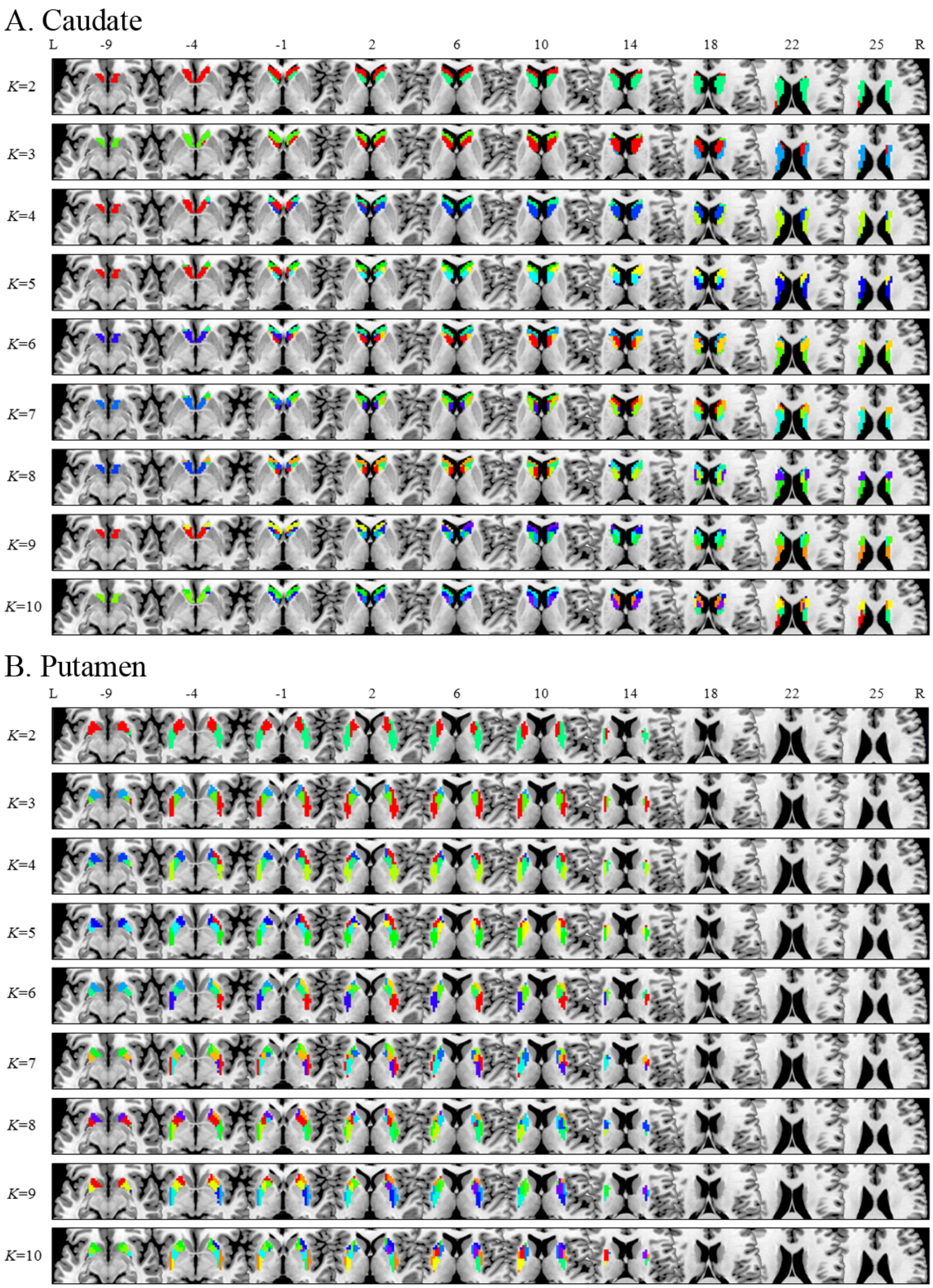

Figure S2. Axial views showing functional connectivity-based parcellation of the caudate (A) and putamen (B) according to different *K* (2–10) cluster solutions. With an increase in *K*, the functional subdivisions of the caudate and putamen were much more detailed and segmented.
