# Supplementary material for: Unravelling the Intrinsic Functional Organization of the Human Striatum: A Parcellation and Connectivity Study Based on Resting-State fMRI
Source: PLoS One. 2014 Sep 9;9(9):e106768. doi: 10.1371/journal.pone.0106768 (PMC4159235; doi:10.1371/journal.pone.0106768)
Supplement: Figure S5 — ROI analysis results for functional connectivity between putamen clusters identified by K = 6 cluster solution and other brain regions, segmented based on the AAL atlas. The x-axis indicates brain regions, and the y-axis indicates the strength of functional connectivity between each cluster as a seed region and other brain regions, as correlation z scores. The red and blue bars respectively indicate the area located in the right and left hemisphere. The red, blue, and purple asterisks respectively indicate that these regions located in the right, left, and bilateral hemisphere had significant functional connectivity with the cluster region under a false discovery rate threshold of q<0.05. (PDF) [file pone.0106768.s005.pdf]

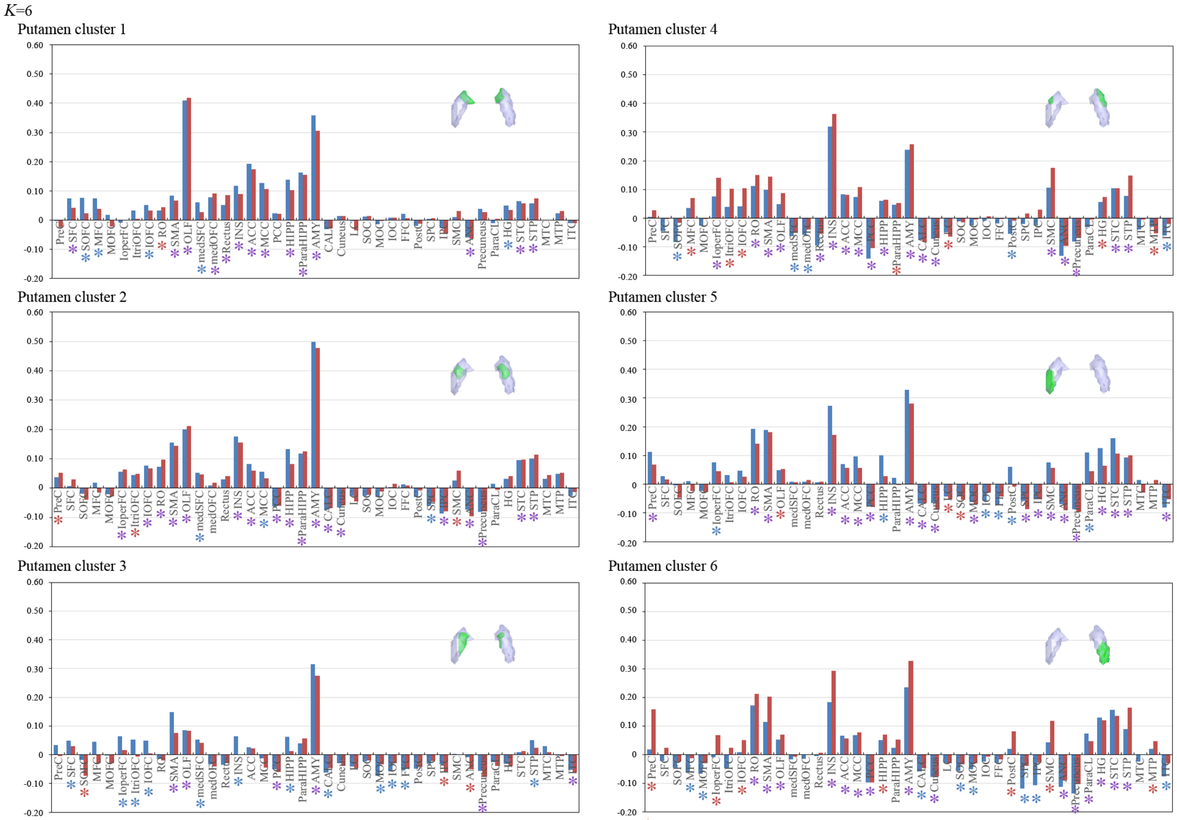

Figure S5. ROI analysis results for functional connectivity between putamen clusters identified by K = 6 cluster solution and other brain regions, segmented based on the AAL atlas. The x-axis indicates brain regions, and the y-axis indicates the strength of functional connectivity between each cluster as a seed region and other brain regions, as correlation z scores. The red and blue bars respectively indicate the area located in the right and left hemisphere. The red, blue, and purple asterisks respectively indicate that these regions located in the right, left, and bilateral hemisphere had significant functional connectivity with the cluster region under a false discovery rate threshold of q<0.05.
