# Supplementary material for: Unravelling the Intrinsic Functional Organization of the Human Striatum: A Parcellation and Connectivity Study Based on Resting-State fMRI
Source: PLoS One. 2014 Sep 9;9(9):e106768. doi: 10.1371/journal.pone.0106768 (PMC4159235; doi:10.1371/journal.pone.0106768)
Supplement: Figure S6 — Functional connectivity maps of the putamen subdivisions in the K = 4 cluster solution. The red and blue areas indicate those showing positive and negative functional correlation with the seed region, respectively. The rostral putamen clusters were positively connected to the areas involved in affective and cognitive processes, while the caudal putamen clusters were positively connected to motor areas. (PDF) [file pone.0106768.s006.pdf]

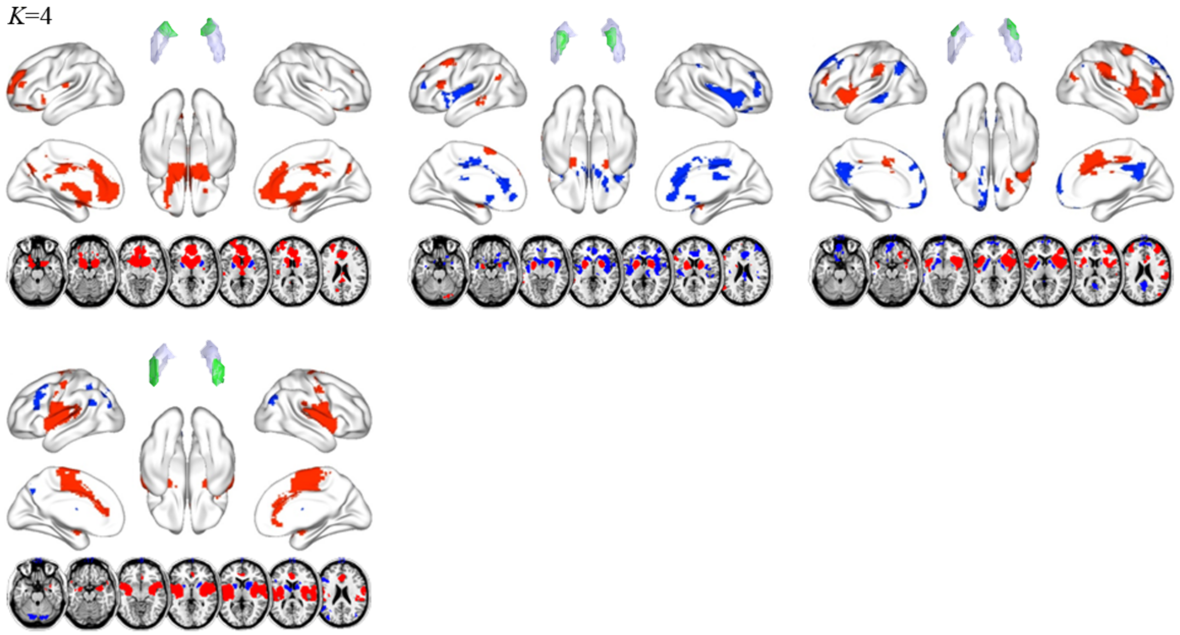

Figure S6. Functional connectivity maps of the putamen subdivisions in the $K = 4$ cluster solution. The red and blue areas indicate those showing positive and negative functional correlation with the seed region, respectively. The rostral putamen clusters were positively connected to the areas involved in affective and cognitive processes, while the caudal putamen clusters were positively connected to motor areas.
