# Supplementary material for: Unravelling the Intrinsic Functional Organization of the Human Striatum: A Parcellation and Connectivity Study Based on Resting-State fMRI
Source: PLoS One. 2014 Sep 9;9(9):e106768. doi: 10.1371/journal.pone.0106768 (PMC4159235; doi:10.1371/journal.pone.0106768)
Supplement: Figure S7 — ROI analysis results for functional connectivity between striatal and thalamic clusters and other regions that belong to CSTC loops. These results were rearranged in order of the strength of the FC between pairs of regions. Part A: Correlations between the caudate clusters and other regions. Part B: Correlations between the putamen clusters and other regions. The x-axis indicates the strength of functional connectivity between the cluster regions and other brain regions as correlation z scores, and the y-axis indicates brain regions, which are rearranged in order of the strength of functional connectivity. For simplicity, we focused on results from bilateral ROI regions. The black asterisks indicate that these regions had significant functional connectivity with the cluster regions under a false discovery rate threshold of q<0.05. (PDF) [file pone.0106768.s007.pdf]

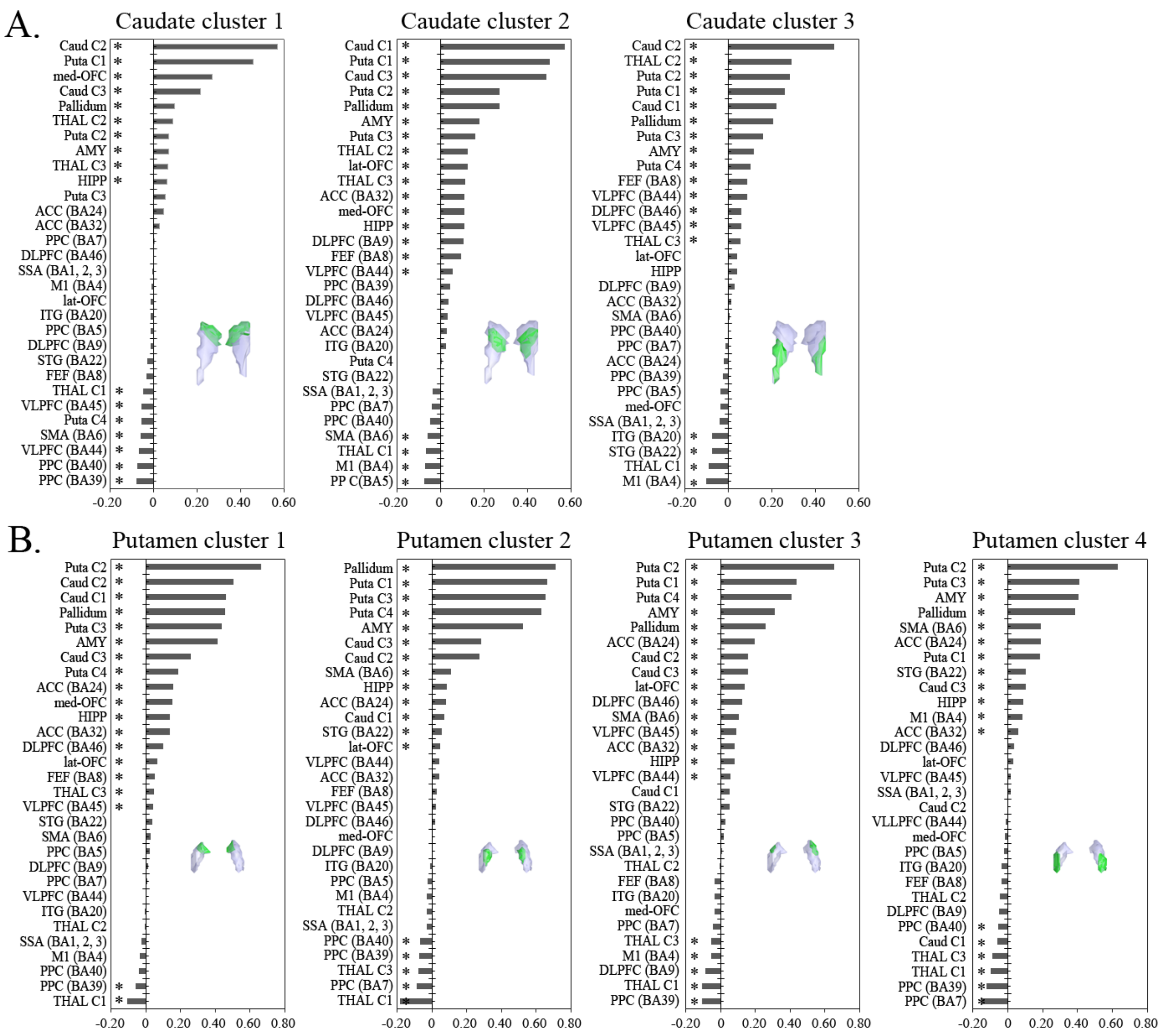

Figure S7. ROI analysis results for functional connectivity between striatal and thalamic clusters and other regions that belong to CSTC loops. These results were rearranged in order of the strength of the FC between pairs of regions. Part A: Correlations between the caudate clusters and other regions. Part B: Correlations between the putamen clusters and other regions. The x-axis indicates the strength of functional connectivity between the cluster regions and other brain regions as correlation z scores, and the y-axis indicates brain regions, which are rearranged in order of the strength of functional connectivity. For simplicity, we focused on results from bilateral ROI regions. The black asterisks indicate that these regions had significant functional connectivity with the cluster regions under a false discovery rate threshold of q<0.05.
