# Supplementary material for: Unravelling the Intrinsic Functional Organization of the Human Striatum: A Parcellation and Connectivity Study Based on Resting-State fMRI
Source: PLoS One. 2014 Sep 9;9(9):e106768. doi: 10.1371/journal.pone.0106768 (PMC4159235; doi:10.1371/journal.pone.0106768)
Supplement: Table S1 — Peak coordinates of connectivity maps with caudate subregions. Coordinates for peak voxels are presented in MNI space. Caud, caudate; OLF, olfactory cortex; SFC, superior frontal cortex; SMC, supramarginal cortex; IPC, inferior parietal cortex; MOFC, middle orbitofrontal cortex; MOC, middle occipital cortex; IOFC, inferior orbitofrontal cortex; MFC, middle frontal cortex; IFC, inferior frontal cortex; medSFC, medial superior frontal cortex; MTC, middle temporal cortex; HIPP, hippocampus; MTC, middle temporal cortex; ANC, angular cortex; ParaCL, paracentral lobule; SFC, superior frontal cortex; MCC, middle cingulate cortex; SMA, supplementary motor area; CALC, calcarine cortex; ACC, anterior cingulate cortex; STC, superior temporal cortex; INS, insula; IOC, inferior occipital cortex; THAL, thalamus; SOC, superior occipital cortex; PreC, precentral cortex, ITC, inferior temporal cortex, SOFC, superior orbitofrontal cortex; FFC, fusiform face cortex; AMY, amygdala; PostC, postcentral cortex; PCC, posterior cingulate cortex; medOFC, medial orbitofrontal cortex. (PDF) [file pone.0106768.s008.pdf]

Table S1. Peak coordinates of connectivity maps with caudate subregions

Regions	Peak (mm) x y z	t-value	z-value
**K=1, Positive connectivity map**			
R Caud/R OLF	9 18 3	36.17	Inf
L Cerebellum	-30 -78 -36	5.16	4.66
L SFC	-27 63 6	5.05	4.57
R Cerebellum	30 -78 -36	4.78	4.37
R Cerebellum	6 -81 -18	4.04	3.77
**K=1, Negative connectivity map**			
R Precuneus	3 -51 72	6.13	5.36
L THAL	-15 -9 -3	4.38	4.05
R SMC/R IPC	57 -39 39	4.36	4.03
**K=2, Positive connectivity map of cluster 1 seed**			
L Caud/L MOFC	-9 18 0	42.46	Inf
L Cunues	3 -84 42	4.81	4.40
L MOC	-45 -87 -9	4.63	4.25
**K=2, Negative connectivity map of cluster 1 seed**			
L Caud/L IOFC	-15 3 18	10.24	7.70
R Caud/R THAL	15 0 18	6.58	5.66
L SMC/L IPC	-57 -57 24	6.04	5.30
R MFC/R IFC	39 9 51	5.82	5.14
R SMC/R IPC	60 -48 27	5.50	4.91
R medSFC	12 36 51	5.48	4.90
R IOFC	42 36 -18	5.45	4.87
L MFC	-42 12 45	5.30	4.77
R MTC	63 -24 -12	5.10	4.62
R HIPP	9 -15 -24	5.00	4.54
L MTC	-63 -24 -15	4.82	4.40
**K=2, Positive connectivity map of cluster 2 seed**			
L Caud	-12 6 12	34.51	Inf
R Cerebellum	15 -78 -27	6.46	5.58
R MFC	42 12 51	5.90	5.20
L ANC/L IPC	-54 -66 39	4.97	4.52
R MTC	63 -15 -15	4.71	4.32
L Cerebellum	0 -51 -42	4.63	4.25
**K=2, Negative connectivity map of cluster 2 seed**			
R Caud/R OLF	15 27 3	8.36	6.74
R ParaCL	3 -45 72	6.59	5.67
L MOC	-33 -93 12	5.73	5.08
R MOC	36 -93 0	5.13	4.64
R MFC	33 -21 69	4.05	3.78
**K=3, Positive connectivity map of cluster 1 seed**			
L Caud/L MOFC	-9 18 0	41.19	Inf
L SFC	-12 69 9	4.15	3.87
**K=3, Negative connectivity map of cluster 1 seed**			
R Caud	12 9 12	10.38	7.77
L Caud	-12 6 15	10.17	7.67
L IOFC	-42 39 -9	7.30	6.12
L SMC	-57 -57 27	6.08	5.33
R SMC	66 -51 27	6.02	5.28
R HIPP	6 -15 -24	5.86	5.17
R MTC	66 -27 -12	5.83	5.15
R MOFC	36 42 -12	5.74	5.08
R MFC	39 12 48	5.58	4.97
L MTC	-57 6 -27	5.46	4.89
L MTC	-63 -24 -15	5.36	4.81
R medSFC	6 48 33	5.34	4.80
L MFC	-42 12 45	5.25	4.73
R MCC	21 -39 33	4.95	4.50
R Cerebellum	18 -78 -30	4.59	4.22
**K=3, Positive connectivity map of cluster 2 seed**			
R Caud	12 12 12	44.78	Inf
R Cerebellum	30 -81 -36	8.26	6.69
R MTC	63 -27 -9	6.94	5.89
L Cerebellum	-30 -78 -36	6.83	5.83
L ANC	-54 -66 39	6.77	5.79
L Precuneus/L MCC	-3 -54 33	5.60	4.99
R HIPP	12 -9 -24	5.38	4.83
L Cerebellum	0 -54 -42	5.31	4.77
R ANC	45 -60 36	5.28	4.75
R MFC	42 15 54	4.90	4.46
**K=3, Negative connectivity map of cluster 2 seed**			
L Caud	-9 21 -3	9.22	7.21
L Caud	-18 3 27	8.13	6.61
R Caud	18 -15 21	6.30	5.48
R ParaCL	3 -45 72	5.83	5.15
R SMA	9 -3 78	4.68	4.29
L CALC	6 -96 -15	4.29	3.98
**K=3, Positive connectivity map of cluster 3 seed**			
L Caud/R Caud	-15 -3 21	41.00	Inf
**K=3, Negative connectivity map of cluster 3 seed**			
L Caud/R ACC	-9 12 6	6.69	5.74
L STC/L INS	-45 -21 6	6.09	5.33
R INS	36 -18 21	6.08	5.33
L Precuneus/L MCC	-9 -42 3	5.87	5.18
L IOC	-36 -81 -9	5.41	4.85
R THAL	6 -12 -3	5.22	4.71
R medSFC/L ACC	15 48 3	5.16	4.66
R IOC	33 -87 -6	5.08	4.60
L MTC	-54 -6 -27	5.00	4.54
L SFC	-15 63 36	4.97	4.52
R SOC	18 -93 36	4.46	4.12
R CALC	18 -48 6	4.42	4.09
L PreC	-36 -27 63	4.36	4.04
L ITC	-66 -24 -18	4.35	4.03
L STC/L MTC	-45 -39 9	4.32	4.01
**K=9, Positive connectivity map of cluster 1 seed**			
R Caud/L Caud	12 21 9	29.86	Inf
L IOC	-36 -84 -9	4.28	3.97
**K=9, Negative connectivity map of cluster 1 seed**			
R Caud	15 21 0	10.76	Inf
L Caud	-15 21 0	8.13	6.62
R SFC	21 60 15	4.81	4.39
**K=9, Positive connectivity map of cluster 2 seed**			
R Caud/R MCC	18 18 12	40.87	Inf
L Caud	-18 18 6	28.10	Inf
L MFC	-21 54 30	6.08	5.33
R medSFC	12 57 18	4.89	4.46
**K=9, Negative connectivity map of cluster 2 seed**			
L Caud/L OLF	-6 21 9	10.86	Inf
R Caud	12 3 15	9.21	7.20
L Caud	-12 0 15	7.94	6.51
L MOC	-39 -93 -9	5.26	4.73
R SOFC	30 66 -6	4.32	4.00
R MFC	54 27 36	4.24	3.94
**K=9, Positive connectivity map of cluster 3 seed**			
L Caud/R Caud	-6 9 6	31.85	Inf
R FFC	21 -51 -12	4.35	4.03
L CALC	-6 -51 3	4.26	3.95
**K=9, Negative connectivity map of cluster 3 seed**			
L Caud	-15 6 15	9.52	7.36
R Caud	15 9 15	9.17	7.18
R Caud	12 18 -9	7.38	6.17
R Caud	18 27 9	7.13	6.02
L Cerebellum	-18 -69 -24	4.83	4.41
L Precuneus	0 -60 66	4.68	4.29
R Cerebellum	21 -93 -30	4.25	3.95
**K=9, Positive connectivity map of cluster 4 seed**			
L Caud	-9 9 18	32.81	Inf
R INS	45 -6 9	6.44	5.57
L HIPP	-24 -42 15	5.49	4.91
L STC	-48 -3 -3	5.09	4.61
R Cerebellum	6 -21 -51	4.20	3.91
**K=9, Negative connectivity map of cluster 4 seed**			
L Caud	-15 6 9	13.95	Inf
L IFC	-48 15 18	6.28	5.46
L MFC	-39 39 27	5.44	4.87
R IFC	54 21 15	5.24	4.72
L IPC	-36 -54 54	5.13	4.64
R Precuneus	24 -48 30	4.93	4.49
R IFC	48 21 -15	4.44	4.10
R SFC	27 66 -9	4.29	3.98
**K=9, Positive connectivity map of cluster 5 seed**			
R Caud/R AMY	12 3 12	30.67	Inf
L IFC	-51 21 12	7.83	6.44
R IOFC	48 21 -15	7.59	6.30
L medSFC	0 39 45	7.44	6.21
L MTC	-66 -33 -9	5.77	5.11
R Cerebellum	15 -81 -27	5.62	5.00
L Cerebellum	-15 -81 -33	5.46	4.88
R MTC	57 -39 -3	5.41	4.85
L SMC	-57 -51 30	4.91	4.47
R ANC	51 -51 30	4.68	4.30
**K=9, Negative connectivity map of cluster 5 seed**			
R ACC/L Caud	6 12 18	11.25	Inf
R INS	39 -9 21	5.88	5.19
L INS	-42 -3 -6	4.80	4.38
L SMC	-48 -24 24	4.39	4.06
**K=9, Positive connectivity map of cluster 6 seed**			
L Caud	-18 6 21	53.45	Inf
R Caud	18 6 24	26.52	Inf
**K=9, Negative connectivity map of cluster 6 seed**			
R Caud	12 9 12	9.88	7.53
R PostC/R Precuneus	24 -45 39	4.86	4.43
R IFC	42 24 24	4.74	4.34
**K=9, Positive connectivity map of cluster 7 seed**			
L Caud/L MOFC	-9 21 3	38.17	Inf
**K=9, Negative connectivity map of cluster 7 seed**			
R Caud	9 12 -6	10.49	7.81
R MCC	9 -12 30	5.97	5.25
R ANC/R Precuneus	30 -42 33	5.37	4.82
L PreC	-48 9 39	5.34	4.80
R Cerebellum	12 -87 -39	5.31	4.77
R INS	45 -12 21	5.11	4.62
L MCC	-12 24 33	4.63	4.25
L PCC	-6 -51 21	4.19	3.90
**K=9, Positive connectivity map of cluster 8 seed**			
L Caud/R Caud	-18 -12 21	38.18	Inf
R THAL/L THAL	3 -24 12	6.64	5.70
**K=9, Negative connectivity map of cluster 8 seed**			
R Caud	21 9 24	6.61	5.69
L Caud	-18 6 18	6.16	5.38
**K=9, Positive connectivity map of cluster 9 seed**			
L Caud/R medOFC	-9 15 -3	49.42	Inf
L Precuneus/L PCC	-6 -63 30	5.94	5.23
L HIPP	-24 -24 -18	4.37	4.04
**K=9, Negative connectivity map of cluster 9 seed**			
L Caud	-6 9 9	6.96	5.91
R IPC	39 -30 60	4.90	4.47
R IFC	57 18 9	4.76	4.36

Coordinates for peak voxels are presented in MNI space. Caud, caudate; OLF, olfactory cortex; SFC, superior frontal cortex; SMC, supramarginal cortex; IPC, inferior parietal cortex; MOFC, middle orbitofrontal cortex; MOC, middle occipital cortex; IOFC, inferior orbitofrontal cortex; MFC, middle frontal cortex; IFC, inferior frontal cortex; medSFC, medial superior frontal cortex; MTC, middle temporal cortex; HIPP, hippocampus; MTC, middle temporal cortex; ANC, angular cortex; ParaCL, paracentral lobule; SFC, superior frontal cortex; MCC, middle cingulate cortex; SMA, supplementary motor area; CALC, calcarine cortex; ACC, anterior cingulate cortex; STC, superior temporal cortex; INS, insula; IOC, inferior occipital cortex; THAL, thalamus; SOC, superior occipital cortex; PreC, precentral cortex, ITC, inferior temporal cortex, SOFC, superior orbitofrontal cortex; FFC, fusiform face cortex; AMY, amygdala; PostC, postcentral cortex; PCC, posterior cingulate cortex; medOFC, medial orbitofrontal cortex.
