# Supplementary material for: Unravelling the Intrinsic Functional Organization of the Human Striatum: A Parcellation and Connectivity Study Based on Resting-State fMRI
Source: PLoS One. 2014 Sep 9;9(9):e106768. doi: 10.1371/journal.pone.0106768 (PMC4159235; doi:10.1371/journal.pone.0106768)
Supplement: Table S2 — Peak coordinates of connectivity maps with putamen subregions. Coordinates for peak voxels are presented in MNI space. Puta, putamen; PreC, precentral cortex; MFC, middle frontal cortex; MCC, middle cingulate cortex; LC, lingual cortex; THAL, thalamus; MOC, middle occipital cortex; ANC, angular cortex; MTC, middle temporal cortex; SMA, supplementary motor area; ACC, anterior cingulate cortex; INS, insula; PosC, postcentral cortex; STC, superior temporal cortex; IPC, inferior parietal cortex; ITC, inferior temporal cortex; IFC, inferior frontal cortex; SMC, suparmarginal cortex; MCC, middle cingulate cortex; MOC, middle occipital cortex; SFC, superior frontal cortex; SOFC, superior orbitofrontal cortex; medOFC, medial orbitofrontal cortex; SPC, superior parietal cortex; medSFC, medial superior frontal cortex. (PDF) [file pone.0106768.s009.pdf]

Table S2. Peak coordinates of connectivity maps with putamen subregions

Regions	Peak (mm) x y z	t-value	z-value
**K=1, Postive connectivity map**			
L Puta/R Puta	-24 9 0	39.58	Inf
R PreC	54 6 48	6.89	5.86
L PreC	-51 3 45	4.38	4.05
L MFC	-30 45 24	4.19	3.9
**K=1, Negative connectivity map**			
L Precuneus	-3 -69 42	6.56	5.65
R LC/L THAL	6 -24 -12	6.44	5.57
R THAL	9 -15 6	6.27	5.46
L MOC/L ANC	-33 -78 36	5.46	4.88
L MTC	-57 -45 -12	5.37	4.82
R ANC	42 -63 42	5.02	4.56
R Cerebellum	0 -21 -36	4.54	4.18
R MTC	66 -36 -12	4.24	3.94
R MFC	39 24 54	4.11	3.83
**K=2, Positive connectivity map of cluster 1 seed**			
L Puta/R Puta	-21 9 0	38.58	Inf
L SMA	0 18 60	5.85	5.16
L MFC/L ACC	-27 48 18	5.79	5.12
R Cerebellum	24 -72 -36	5.61	4.99
R Cerebellum	36 -63 -27	5.31	4.77
L PreC	-45 9 48	5.20	4.69
L Cerebellum	-39 -69 -27	5.16	4.66
**K=2, Negative connectivity map of cluster 1 seed**			
L Puta/L INS	-33 -6 3	10.27	7.72
R Puta	33 -3 6	9.36	7.28
R PostC	42 -24 63	4.25	3.95
**K=2, Positive connectivity map of cluster 2 seed**			
R Puta/R INS	30 0 3	38.45	Inf
L Puta/L STC	-30 -3 3	36.58	Inf
L SMA/R SMA	-3 -6 66	8.66	6.91
R PreC	51 3 48	5.90	5.20
L PreC	-42 -6 54	5.12	4.63
L Cerebellum	-6 -60 -15	4.42	4.09
**K=2, Negative connectivity map of cluster 2 seed**			
L Puta/L Caud	-18 15 -6	10.83	Inf
R Caud	18 18 -3	9.69	7.44
L Precuneus	-3 -72 36	7.41	6.19
R Cerebellum	18 -78 -33	7.19	6.05
L Cerebellum	-6 -27 -18	6.42	5.56
L IPC	-30 -75 45	6.22	5.42
L ITC	-60 -57 -18	5.22	4.71
L MFC	-36 9 63	4.96	4.51
L THAL	-3 -18 3	4.64	4.26
L Cerebellum	-9 -51 -48	4.63	4.25
R MTC	69 -36 -9	4.40	4.07
L MTC	-60 -36 -12	4.27	3.96
**K=3, Positive connectivity map of cluster 1 seed**			
L Puta/L THAL	-18 15 -3	44.24	Inf
R MFC	33 45 36	6.16	5.38
R Cuneus	15 -69 36	5.57	4.96
R Puta/R INS	36 -9 0	5.19	4.69
L Precuneus	-6 -72 36	4.85	4.42
L INS	-39 -18 15	4.83	4.41
R STC	48 -33 12	4.11	3.83
**K=3, Negative connectivity map of cluster 1 seed**			
R Puta	24 6 6	10.28	7.72
L Puta	-24 3 9	9.75	7.47
L ANC	-48 -63 42	4.17	3.88
**K=3, Positive connectivity map of cluster 2 seed**			
L Puta	-24 6 0	47.98	Inf
R Puta	24 9 3	33.00	Inf
L SMA	-3 18 63	5.73	5.08
L MFC	-42 6 51	5.05	4.58
L IFC	-45 18 18	4.79	4.38
**K=3, Negative connectivity map of cluster 2 seed**			
R Puta/L Puta	33 -9 3	13.68	Inf
L Puta	-33 -9 3	9.66	7.43
R THAL	9 -21 6	6.13	5.36
R MFC	33 42 33	5.10	4.62
R INS	57 3 3	4.91	4.47
L Cuneus	-18 -63 24	4.79	4.38
R SMC/R IPC	48 -42 39	4.07	3.80
**K=3, Positive connectivity map of cluster 3 seed**			
R Puta/R INS	33 -12 3	38.71	Inf
L Puta/L INS	-30 -3 3	28.02	Inf
L MCC/L SMA	0 -3 48	9.67	7.43
L MFC	-30 42 30	4.35	4.03
**K=3, Negative connectivity map of cluster 3 seed**			
L Puta/L THAL	-21 9 3	10.36	7.76
R Puta	21 6 6	9.87	7.53
L Cerebellum	-18 -84 -33	7.67	6.35
L IPC	-33 -75 42	7.35	6.15
L Cerebellum	-6 -27 -18	6.41	5.55
L MFC	-42 12 54	5.93	5.22
L MCC/L SMA	-12 -45 33	5.88	5.19
L MTC	-48 -42 -6	5.41	4.85
L MOC/L ANC	39 -81 36	5.17	4.67
R MTC	69 -36 -9	4.35	4.03
**K=4, Positive connectivity map of cluster 1 seed**			
L Puta/L ACC	-18 15 -3	46.65	Inf
R Puta/R INS	33 -6 0	5.72	5.07
R MFC	33 45 36	5.35	4.80
L Precuneus	-6 -72 36	5.33	4.79
R Cuneus	15 -69 33	5.00	4.54
L INS	-36 -15 18	4.67	4.28
**K=4, Negative connectivity map of cluster 1 seed**			
R Puta	24 3 6	9.52	7.36
L Puta	-24 0 6	7.07	5.98
**K=4, Positive connectivity map of cluster 2 seed**			
L Puta	-24 6 0	47.38	Inf
R Puta	24 6 6	31.40	Inf
L SMA	-3 18 66	6.38	5.53
L IFC	-54 24 18	5.22	4.71
L MFC	-42 12 48	5.00	4.54
R Cerebellum	24 -84 -24	4.83	4.41
L MTC	-51 -42 -6	4.79	4.38
L ANC	-54 -60 24	4.02	3.76
**K=4, Negative connectivity map of cluster 2 seed**			
R Puta	33 -9 3	11.43	Inf
L INS	-33 -6 6	8.72	6.94
R THAL	9 -21 6	6.06	5.31
L MFC	-42 48 24	4.95	4.50
R SMC	48 -42 39	4.49	4.14
**K=4, Positive connectivity map of cluster 3 seed**			
R Puta	27 15 3	46.32	Inf
L INS	-27 15 9	18.16	Inf
R SMA/R MCC	6 9 51	6.65	5.71
R SMC	66 -30 42	6.45	5.58
L MFC	-42 48 27	6.29	5.47
L SMC	-60 -27 21	5.89	5.20
L MCC	-15 -27 33	5.13	4.64
R MOC	42 -78 24	4.61	4.23
L Precuneus	-36 -6 51	4.47	4.13
**K=4, Negative connectivity map of cluster 3 seed**			
L Puta	-21 6 0	7.78	6.41
L Rectus	-3 60 -18	6.81	5.81
L MCC	0 -45 33	6.49	5.60
R Puta	27 -3 3	5.87	5.18
L ANC	-51 -63 33	5.67	5.04
R Cerebellum	27 -87 -42	5.31	4.77
R SFC	21 36 51	5.27	4.74
L MFC	-39 21 48	5.00	4.54
L MTC	-63 -30 -9	4.93	4.49
L SOFC	-12 27 -24	4.30	3.99
R ANC	54 -69 36	4.28	3.97
**K=4, Positive connectivity map of cluster 4 seed**			
R Puta/R INS	33 -12 3	39.08	Inf
L Puta/L INS	-30 -3 3	31.05	Inf
L SMA	-6 -6 72	8.55	6.85
L PreC	-39 -18 51	5.30	4.77
R PreC	51 -6 48	5.02	4.56
**K=4, Negative connectivity map of cluster 4 seed**			
R Puta	24 15 6	9.10	7.14
L Puta/L THAL	-21 9 6	8.65	6.90
R ANC	39 -81 36	6.34	5.51
L Cerebellum	-18 -84 -30	6.11	5.35
R Cerebellum	12 -84 -30	6.01	5.27
L MOC	-33 -72 36	5.58	4.97
L MFC	-33 9 63	5.32	4.78
L Precuneus	-3 -72 51	4.91	4.47
L ITC	-60 -57 -18	4.56	4.20
R IFC	48 24 27	4.55	4.19
L SMC	-54 -54 27	4.52	4.17
**K=6, Positive connectivity map of cluster 1 seed**			
L Puta/L ACC	-18 15 -6	48.52	Inf
R Puta/R INS	33 -6 3	6.11	5.34
R MFC	30 45 36	5.50	4.91
R INS	42 -15 21	5.33	4.79
R Cuneus	15 -69 36	5.07	4.60
L Precuneus	-6 -72 36	4.98	4.53
L STG/L INS	-48 -18 9	4.09	3.82
R SFC	27 54 9	4.05	3.79
**K=6, Negative connectivity map of cluster 1 seed**			
R Puta	24 6 6	10.57	Inf
L Puta	-24 6 3	8.24	6.68
**K=6, Positive connectivity map of cluster 2 seed**			
R Puta	27 9 -3	38.01	Inf
L Puta	-24 6 -6	36.73	Inf
R IFC	45 21 24	5.31	4.77
R Rectus	3 45 -24	4.48	4.13
**K=6, Negative connectivity map of cluster 2 seed**			
R Puta/R INS	27 21 3	9.62	7.41
L Puta/L INS	-15 18 -3	8.75	6.96
R Puta/R INS	33 -9 3	7.27	6.11
L ACC	3 36 21	5.24	4.72
R MFC	27 54 30	4.33	4.01
**K=6, Positive connectivity map of cluster 3 seed**			
L Puta	-21 6 9	50.78	Inf
R Puta	21 9 9	21.11	Inf
R Cerebellum	27 -69 -30	5.91	5.21
L SMA	-3 21 60	5.74	5.09
**K=6, Negative connectivity map of cluster 3 seed**			
R Puta	21 12 -9	11.80	Inf
L MCC	-12 -18 42	5.42	4.85
L medOFC	-12 45 -6	4.30	3.99
R MFC	42 42 3	4.22	3.93
**K=6, Positive connectivity map of cluster 4 seed**			
R Puta/R IFC	27 18 3	49.43	Inf
L INS	-27 15 9	18.90	Inf
R SMC	63 -30 42	6.98	5.93
R MCC	6 18 42	6.41	5.55
L SMC	-63 -30 24	5.98	5.26
L MFC	-42 48 27	5.88	5.19
R MCC	12 -33 42	5.49	4.91
R SFC	24 0 57	5.35	4.81
L MCC	-15 -27 36	5.01	4.55
L PreC	-36 -6 51	4.62	4.25
R SPC	45 -42 57	4.50	4.15
**K=6, Negative connectivity map of cluster 4 seed**			
R Puta	27 3 -6	8.16	6.63
L Puta	-21 6 0	7.71	6.37
L SFC/L medSFC	-15 45 48	6.46	5.59
L MCC	0 -45 33	5.63	5.01
L ANC	-51 -63 33	5.54	4.94
L MTC	-63 -30 -9	5.26	4.73
R Cerebellum	27 -84 -39	5.18	4.68
L MFC	-39 21 45	4.99	4.53
R SFC	24 36 48	4.79	4.37
**K=6, Positive connectivity map of cluster 5 seed**			
L Puta/L INS	-30 -3 3	57.00	Inf
L SMA/L MCC	-3 -6 66	6.98	5.92
R INS	48 6 -9	5.17	4.67
L PreC	-42 -12 51	5.08	4.60
**K=6, Negative connectivity map of cluster 5 seed**			
R Puta	24 15 9	7.51	6.25
L Cerebellum	-30 -75 -27	4.71	4.32
**K=6, Positive connectivity map of cluster 6 seed**			
R Puta/R INS	30 -3 0	47.44	Inf
L INS	-27 -24 12	5.82	5.15
L STC	-63 -6 3	5.78	5.12
R PreC	36 -12 66	5.36	4.81
**K=6, Negative connectivity map of cluster 6 seed**			
L Puta	-24 6 -6	6.95	5.90
R Puta	24 9 -6	5.64	5.02
L IFC	-48 27 27	5.47	4.89
R Cerebellum	6 -81 -27	5.22	4.70
L MFC	-27 42 3	4.85	4.42
L Precuneus	-3 -72 51	4.67	4.28
L IPC	-27 -75 48	4.57	4.20
L MFC	-30 15 63	4.41	4.08

Coordinates for peak voxels are presented in MNI space. Puta, putamen; PreC, precentral cortex; MFC, middle frontal cortex; MCC, middle cingulate cortex; LC, lingual cortex; THAL, thalamus; MOC, middle occipital cortex; ANC, angular cortex; MTC, middle temporal cortex; SMA, supplementary motor area; ACC, anterior cingulate cortex; INS, insula; PosC, postcentral cortex; STC, superior temporal cortex; IPC, inferior parietal cortex; ITC, inferior temporal cortex; IFC, inferior frontal cortex; SMC, suparmarginal cortex; MCC, middle cingulate cortex; MOC, middle occipital cortex; SFC, superior frontal cortex; SOFC, superior orbitofrontal cortex; medOFC, medial orbitofrontal cortex; SPC, superior parietal cortex; medSFC, medial superior frontal cortex.
